# Molecular Structure, Matrix-Isolation IR Spectrum and UV-Induced Transformations of 2-Amino-5-(4-Methoxyphenyl)-1,3,4-Oxadiazole

**DOI:** 10.3390/molecules30163444

**Published:** 2025-08-21

**Authors:** İsa Sıdır, Susy Lopes, Rui Fausto, A. J. Lopes Jesus

**Affiliations:** 1 Department of Physics, Faculty of Sciences and Letters, Bitlis Eren University, Bitlis 13000, Türkiye; 2 University of Coimbra, CQC–IMS, Department of Chemistry, 3004–535 Coimbra, Portugal; susylopes@qui.uc.pt (S.L.); rfausto@ci.uc.pt (R.F.); 3 Spectroscopy@IKU, Faculty of Sciences and Letters, Department of Physics, Istanbul Kultur University, Ataköy Campus, Bakırköy 34156, Istanbul, Türkiye; 4 University of Coimbra, CQC-IMS, Faculty of Pharmacy, 3000-548 Coimbra, Portugal

**Keywords:** 1,3,4-oxadiazoles, conformers and tautomers, matrix-isolation infrared spectroscopy, UV-induced photochemistry, quantum chemical calculations

## Abstract

The photochemistry of 1,3,4-oxadiazoles remains poorly understood, despite their recognized importance in medicinal chemistry and materials science. In this work, we report a detailed matrix-isolation study of 2-amino-5-(4-methoxyphenyl)-1,3,4-oxadiazole, combining low-temperature infrared spectroscopy with broadband UV photolysis and quantum chemical calculations. Theoretical analysis predicts the gas-phase molecule to exist exclusively as the amino tautomer, populating two nearly isoenergetic conformers (anti and syn) defined by the relative orientation of the amino and methoxy groups. Experimental IR spectra of the compound isolated in Ar and Xe matrices at 15 K confirm sole trapping of the amino tautomer. Annealing of the Xe matrix to the highest achievable temperature induced no detectable spectral changes, consistent with the predicted isoenergetic character of the conformers. Upon broadband UV irradiation (*λ* > 200 nm), the compound undergoes ring opening through N−N and C−O bond cleavages, paralleling the behavior of unsubstituted 1,3,4-oxadiazole system. Isocyanates emerge as the predominant photoproducts from these photochemical pathways. Additionally, spectroscopic evidence supports an alternative reaction pathway involving early-stage amino−imino tautomerization, followed by ring-opening of the imino tautomer through isocyanic acid extrusion, leading to the formation of a nitrilimine intermediate. This reactive species subsequently photorearranges into a carbodiimide via a diazirine-mediated pathway. All photoproducts were unambiguously identified through their distinct IR signatures, supported by quantum chemical calculations and reference data from structurally related systems. These findings provide unprecedented insight into the photochemical behavior of substituted 1,3,4-oxadiazoles and unveil new reaction pathways modulated by substituent effects, expanding the understanding of their photoreactivity.

## Simple Summary

Despite the versatility of oxadiazoles in medicinal chemistry and materials science, their photochemical behavior remains poorly characterized. We investigated the UV-driven reactions of 2-amino-5-(4-methoxyphenyl)-1,3,4-oxadiazole isolated in Ar and Xe matrices. Both in the gas phase and in low-temperature matrices, the compound exists exclusively as the amino tautomer, adopting nearly isoenergetic anti and syn conformers distinguished by the relative orientation of the amino and methoxy groups. UV exposure of the matrix-isolated compound triggers three competitive pathways: two involve oxadiazole N–N or C–O bond cleavage, yielding isocyanate derivatives as primary products, in line with the photochemistry of unsubstituted 1,3,4-oxadiazole. The third pathway initiates with amino–imino tautomerization, followed by ring-opening of the imino tautomer through isocyanic acid extrusion, leading to the formation of a nitrilimine intermediate. This intermediate undergoes a photorearrangement via diazirine to form a carbodiimide. All photoproducts were identified through their characteristic infrared signatures, supported by quantum chemical calculations and comparison with reference systems. These findings provide new insights into the photochemistry of oxadiazoles and demonstrate how substituent effects can steer their reactivity.

## 1. Introduction

**Figure 1:**
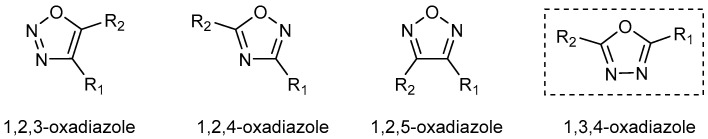
The four isomeric forms of oxadiazole, with R_1_ and R_2_ denoting substituents.

Oxadiazoles are heterocyclic compounds characterized by a five-membered ring containing one oxygen and two nitrogen atoms. The arrangement of these heteroatoms within the ring gives rise to four distinct positional isomers, as represented in Figure 1. Among these, 1,3,4-oxadiazole has garnered particular attention, especially in medicinal chemistry, due to its broad spectrum of therapeutic activities [1,2,3,4,5,6,7,8,9,10,11]. This diversity of bioactivity arises from the unique electronic and structural characteristics of the 1,3,4-oxadiazole core, which enables it to act as a bioisostere for functional groups such as carboxylic acids, esters, and amides [12,13,14,15]. By replacing these groups in drug candidates, the oxadiazole ring can enhance metabolic stability, modulate lipophilicity, and improve pharmacokinetic properties [15,16], making it a valuable motif in drug design. Reflecting this broad therapeutic potential, several 1,3,4-oxadiazole derivatives have been approved or have reached late-stage clinical development [2,11,13,17,18].

In the field of materials science, 1,3,4-oxadiazole derivatives have also emerged as prominent electron-transporting materials in organic light-emitting diodes (OLEDs) [19,20,21,22]. This interest arises from their strong electron-withdrawing ability, which lowers the energy of the lowest unoccupied molecular orbital (LUMO), promoting efficient electron injection and transport from the cathode to the emissive layer.

Due to their broad utility in medicinal and materials science applications, a variety of synthetic strategies have been reported for the preparation of 1,3,4-oxadiazoles. These include oxidative cyclization, cyclodesulfurization, cyclodehydration, condensation reactions, and the Huisgen reaction [4,23,24].

**Scheme 1:**
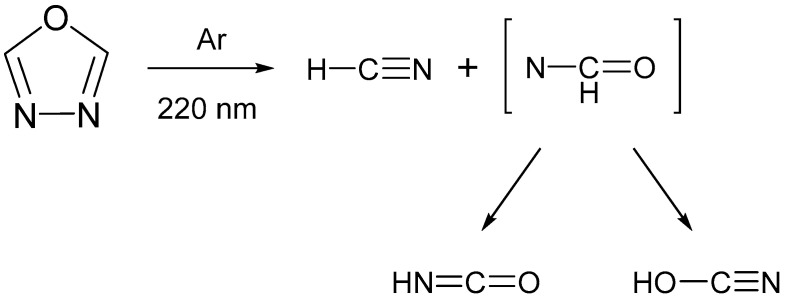
UV-induced (λ = 200 nm) cleavage of the formally single C–O and N–N bonds in 1,3,4-oxadiazole, isolated in a low temperature Ar matrix (10 K), resulting in the formation of hydrogen cyanide (HCN) as well as isocyanic (HNCO) and cyanic (HOCN) acids. Adapted from ref. [26]. The species in square brackets is a nitrene intermediate, which promptly rearranges via hydrogen atom transfer, yielding either HNCO or HOCN.

Despite this considerable synthetic and application-driven interest, the photochemical behavior of functionalized 1,3,4-oxadiazoles remains comparatively underexplored [25,26,27]. A distinctive feature of these heterocycles is their aromatic character combined with the presence of labile C–O and N–N single bonds, making them susceptible to bond cleavage upon UV irradiation. In a study by Keresztes et al. [26], the photochemistry of unsubstituted 1,3,4-oxadiazole (R_1_, R_2_ = H) was investigated under cryogenic conditions (Ar, 10 K). Upon UV irradiation at *λ* = 220 nm, the compound underwent photofragmentation leading to the formation of hydrogen cyanide (HCN), isocyanic acid (HNCO), and cyanic acid (HOCN), see Scheme 1. These photoproducts were trapped in the matrix as hydrogen-bonded complexes, namely HCN···HNCO and HCN···HOCN, as revealed by infrared spectroscopy supported by quantum chemical calculations. The rigid Ar matrix exerted a pronounced cage effect, stabilizing the complexes and preventing their dissociation.

More recently, the photochemistry of a pyridyl-substituted 1,3,4-oxadiazole-thione isolated in an Ar matrix was studied in our laboratory [27]. Upon applying broadband UV irradiation (*λ* > 235 nm), the matrix-isolated compound underwent photofragmentation via carbonyl sulfide (OCS) extrusion, generating a nitrile imine intermediate. This reactive species is subsequently rearranged through a diazirine-mediated pathway, yielding carbodiimide and cyanamide as stable photoproducts.

Building on these findings, in the present work, we extend these studies to 2-amino-5-(4-methoxyphenyl)-1,3,4-oxadiazole (R_1_ = NH_2_ and R_2_ = 4-methoxyphenyl), aiming to investigate how electronic effects introduced by the methoxyphenyl and amino substituents influence the photochemical pathways under matrix isolation conditions. In particular, the amino substituent positioned vicinal to a ring heteroatom enables the possibility of amino–imino tautomerization, which may significantly influence the reaction pathways and govern the nature of the photoproducts formed. Through a combination of UV-induced photolysis and IR spectroscopy, supported by quantum chemical calculations, we seek to gain deeper insight into the structure–reactivity relationships governing the photochemistry of substituted oxadiazoles.

## 2. Results and Discussion

### 2.1. Isomeric Forms of 2-Amino-5-(4-Methoxyphenyl)-1,3,4-Oxadiazole ***1***

**Figure 2:**
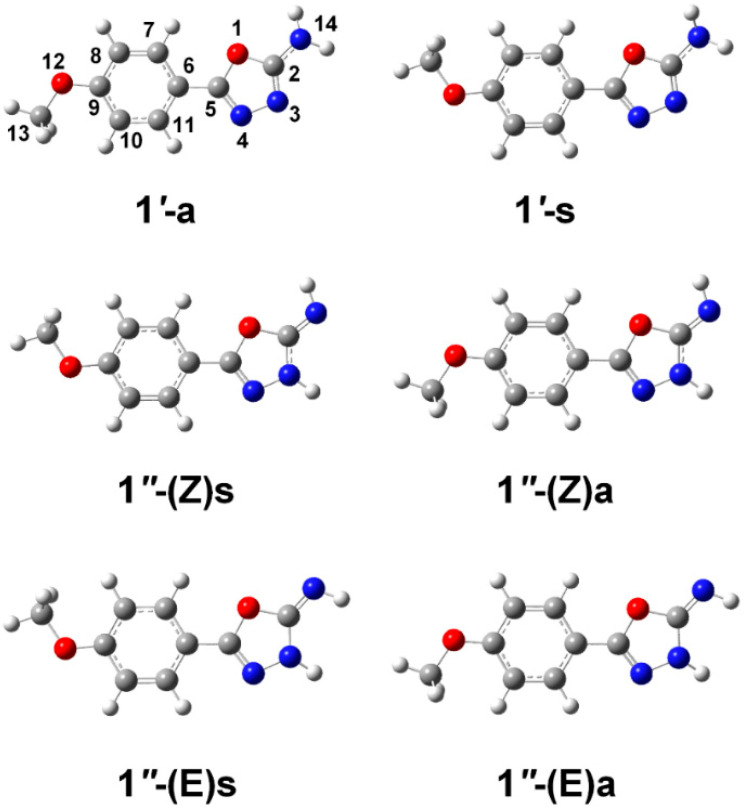
B3LYP/6-311++G(d,p) fully optimized geometries of the isomeric forms of 2-amino-4-(methoxyphenyl)-1,3,4-oxadiazole **1**. The isomers are labeled as **1′** (amino tautomer) and **1″** (imino tautomer), followed by the letters **a** (anti) or **s** (syn), indicating the relative orientation of the methoxy group with respect to the amino or imino group. For the isomers exhibiting the **1″** tautomeric form, the **Z** or **E** configuration around the exocyclic C=N bond is also specified.

2-Amino-5-(4-methoxyphenyl)-1,3,4-oxadiazole **1** can exist as either amino or imino tautomers, abbreviated as **1′** and **1″**, respectively (see Figure 2). The latter arises from proton migration from the amino group to the adjacent ring nitrogen (N3), accompanied by a reorganization of the π-electron system. Tautomer **1′** adopts two conformers, **1′-a** and **1′-s**, which differ in the relative spatial orientation of the amino and methoxy substituents. In **1′-a**, these groups are arranged in an *anti* (**a**) fashion, pointing to opposite sides of the molecular plane, whereas in **1′-s**, they are positioned in a syn (s) configuration, pointing toward the same side. Tautomer **1″** displays greater structural diversity with four possible isomeric forms: **1″-**(**Z**)**s**, **1″-**(**Z**)**a**, **1″-**(**E**)**s**, and **1″-**(**E**)**a**. These isomers arise from the combination of (i) **Z**/**E** configurations around the exocyclic C=N bond and (ii) *syn*/*anti* orientations between the methoxy and imino groups.

Table 1 presents the B3LYP/6-311G(d,p) calculated relative energies, estimated from Boltzmann populations at 298.15 K and dipole moments for the six isomeric species of **1** (cartesian coordinates of the optimized geometries as provided as Supplementary Material). The results indicate that the **1′** isomers have significantly lower energies than their **1″** counterparts, with minimum energy gaps of 17, 16 and 14 kJ mol^−1^, depending on whether electronic, zero-point corrected, or Gibbs free energies are considered, respectively. This marked stability difference originates from fundamental electronic structure considerations. The **1′** isomers maintain π-conjugation throughout the oxadiazole ring system, while proton transfer to N3 in the **1″** forms partially disrupts this delocalization, significantly reducing molecular stabilization. The calculated Boltzmann populations at 298.15 K reflect this significant energetic disparity, yielding a **1′**:**1″** ratio of approximately 99.8:0.2. This result indicates that under isolated conditions, such as those encountered in cryogenic matrix isolation experiments, tautomer **1′** would be the only observable form of the compound.

The B3LYP/6-311++G(d,p) calculations reveal a very small energy difference (<0.4 kJ mol^−1^) between the **1′-a** and **1′-s** conformers, with **1′-a** being slightly favored. To assess the consistency of this result, additional calculations were performed using two complementary approaches: (i) B3LYP/aug-cc-pVTZ, which employs a more complete and flexible basis set within the same DFT framework, and (ii) MP2/6-311++G(d,p), a correlated wavefunction-based method capable of capturing dispersion and electron correlation more accurately. The obtained results have been included in Table 1. The B3LYP calculations with the larger basis sets maintained both the stability order and energy separation magnitude. MP2 electronic energies also preserved this trend. However, inclusion of zero-point energy, thermal corrections and entropic effects led to a reversal of the energetic preference, now favoring **1′-s**, though the energy difference between the two conformers remain very small, below 0.5 kJ mol^−1^. These nearly isoenergetic conformers result in approximately equal Boltzmann gas-phase populations. In the context of the matrix isolation experiments, this implies that the gaseous compound before the matrix deposition should contain almost equal amounts of each conformer.

**Figure 3:**
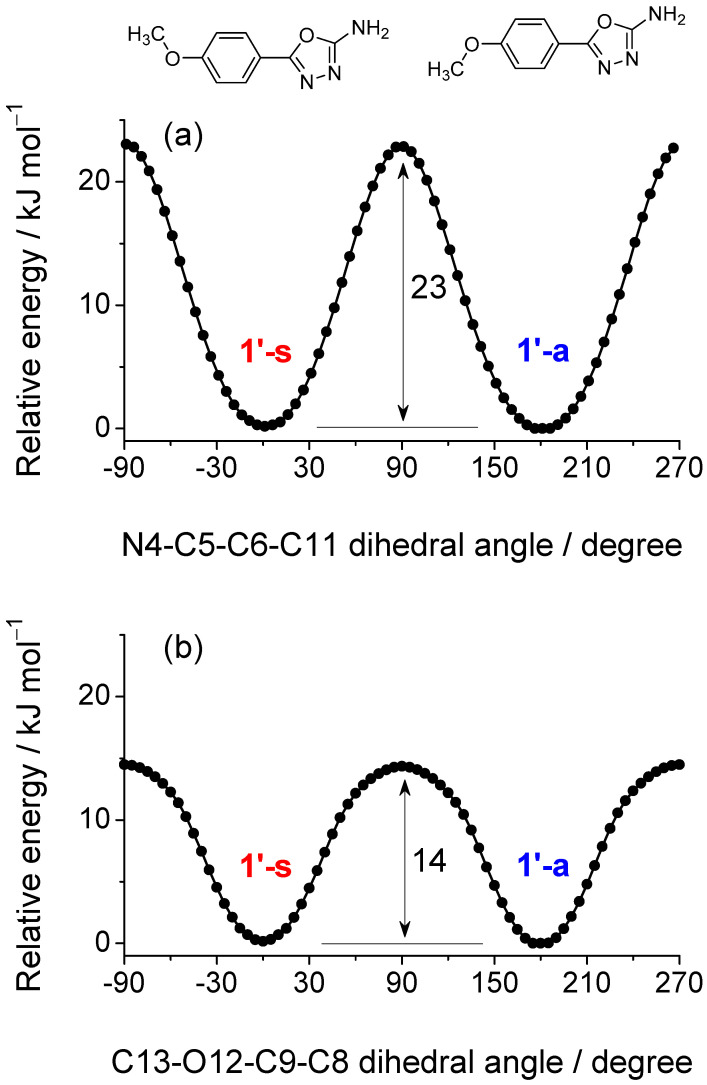
Energy barriers for the interconversion between conformers **1′-s** and **1′-**a (shown at the top of the figure), calculated via two distinct pathways: (**a**) rotation around the central inter-ring C5–C6 bond and (**b**) rotation of the methoxy group. The electronic energy profiles were obtained from relaxed potential energy scans, in which the torsional coordinate was incrementally varied while all other internal degrees of freedom were optimized at each step. Barriers of 23 and 14 kJ mol^−1^ were obtained for these two processes, respectively; the latter reduces to 13 kJ mol^−1^ when zero-point energy corrections are included.

Interconversion between **1′-a** and **1′-s** can occur via two distinct pathways: (i) rotation of the methoxy group around the C9−O12 bond and (ii) torsion around the C5−C6 bond, which connects the phenyl ring to the 1,3,4-oxadiazole core. Potential energy profiles calculated at the B3LYP/6-311++G(d,p) level (Figure 3) reveal that the energy barrier for methoxy rotation, 14 kJ mol^−1^ based on electronic energies, or 13 kJ mol^−1^ when zero-point energy corrections are included, is significantly lower than that associated with the torsion around the C5−C6 bond (23 kJ mol^−1^). These results suggest that interconversion between the two conformers is more easily achieved via methoxy rotation, whereas rotation of the entire aromatic substituent is less favorable.

Under matrix-isolation conditions, the methoxy rotational barrier, though lower than the C5−C6 torsional barrier, remains sufficiently high to prevent conformational cooling, i.e., relaxation to the most stable conformer, whether it be **1′-a** or **1′-s**, during the matrix deposition at 15 K [28,29,30,31]. This is supported by previous studies from our group on structurally analogous systems exhibiting OCH_3_ rotamerism [32,33,34]. For instance, 5- and 6-methoxyindole [32,33], exist as two conformers, differing in the relative orientation of the methoxy and NH groups, pointing either in the same direction (syn) or in opposite directions (anti). Despite the relatively low energy barrier for methoxy rotation in the direction of conformational decay (6–9 kJ mol^−1^), both the lower-energy and higher-energy conformers were successfully trapped and spectroscopically characterized in Ar and Xe matrices at 16–20 K. Similarly, in 4-methoxybenzaldehyde [34], a barrier of ~10 kJ mol^−1^ allowed observation of the two stable rotamers in both noble-gas matrices (10–30 K). Given that the methoxy rotamerization barrier in the present system is even higher (13–14 kJ mol^−1^), we expect efficient trapping and preservation of **1′-a** and **1′-s** conformers in an as-deposited matrices.

### 2.2. Matrix Isolation Spectrum of the Freshly Deposited Matrices

**Figure 4:**
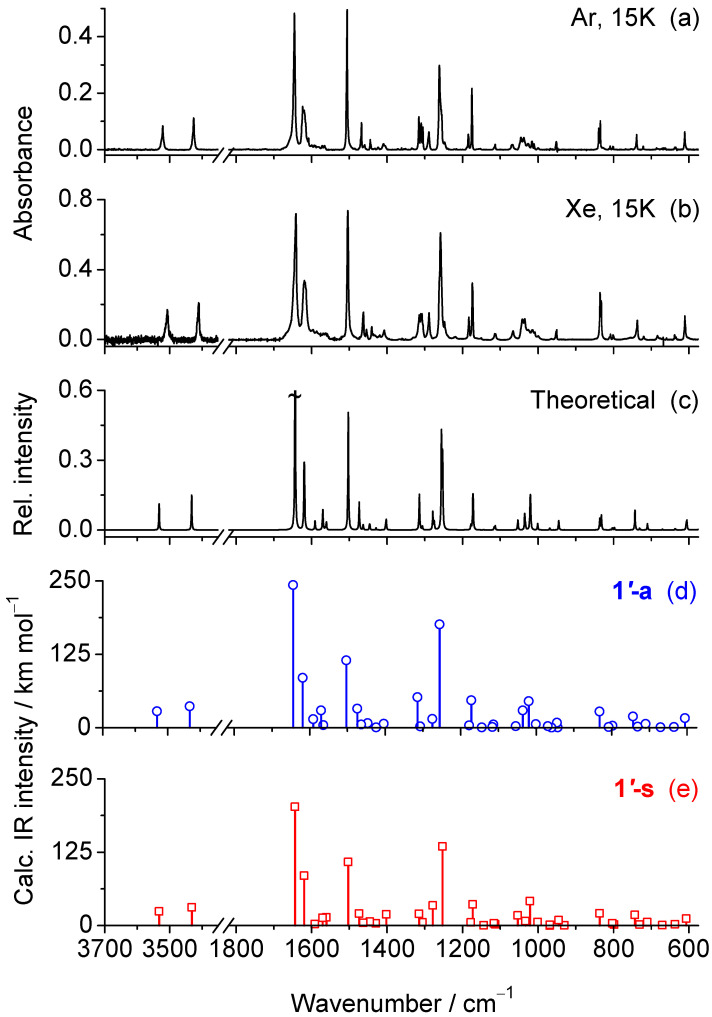
(**a**,**b**) Experimental IR spectrum of 2-amino-5-(4-methoxyphenyl)-1,3,4-oxadiazole **1** isolated in an Ar and Xe matrices at 15 K. (**c**) Theoretical gas-phase IR spectrum of **1**, simulated as the average of the B3LYP/6-311++G(d,p) spectra calculated for the **1′-a** and **1′-s** conformers, i.e., assuming a 1:1 ratio. The spectrum was simulated with Lorentzian band shapes with a full width at half maximum (FWHM) of 2 cm^−1^, centered at the scaled wavenumbers. The strongest band predicted at 1645 cm^−1^ is truncated. (**d**,**e**) Stick spectra extracted from the vibrational frequency calculations showing the scaled wavenumbers and unscaled IR intensities calculated for conformers **1′-a** (**d**) and **1′-s** (**e**).

The infrared spectra of monomers of compound **1** isolated in Ar and Xe matrices at 15 K are shown in Figure 4a and Figure 4b, respectively, covering the 3700–3350 cm^−1^ and 1800–575 cm^−1^ regions. As can be seen, both experimental spectra agree to each other and also exhibit excellent agreement with the gas-phase theoretical spectrum calculated at the B3LYP/6-311++G(d,p) level for the isolated molecule (Figure 4c), which was simulated by averaging the vibrational data obtained for conformers **1′-s** and **1′-**a (Figure 4d,e). This good correspondence confirms that the monomers trapped in the as-deposited cryogenic matrices adopt a **1′** tautomeric structure, in full agreement with the theoretical predictions. Also, in consonance with the theoretical calculations, the **1″** isomers can be confidently excluded from the matrix composition, as their predicted IR spectra show no agreement with the experimental data (see Figure S1). In particular, the strongest and very characteristic absorption band predicted for these isomers in the 1750–1730 cm^−1^ interval (see Table S1) and assigned to the stretching vibration of the exocyclic C=N bond [ν(C=N)], is absent in the experimental spectra.

Tautomer **1′** adopts a molecular structure belonging to the *C*_1_ symmetry point group. As a result, all 63 fundamental vibrational modes are predicted to be infrared-active. Assignment of the observed bands to the normal modes calculated for **1′-a** and **1′-s**, is given in Table 2. The high frequency region of the experimental spectra exhibits two weak bands at 3521 and 3426 cm^−1^ (Ar) or 3507 and 3411 (Xe). These features are assigned to the ν(NH_2_)_as_ and ν(NH_2_)_s_ stretching modes, respectively. The positions observed in the Ar matrix closely match the calculated frequencies at 3525 and 3425 cm^−1^, while a red shift of 14–15 cm^−1^ is observed in the more polarizable Xe matrix, reflecting stronger host-guest interactions. In the fingerprint region, the spectra are dominated by the bands at 1645, 1624, 1505, 1261, 1175 cm^−1^ (Ar) or at 1642, 1619, 1504, 1258 and 1174 cm^−1^ (Xe). The corresponding theoretical peaks for **1′-a**/**1′-s** are predicted at 1644/1644 [ν(C2=N3)–ν(C2−N14); δ(NH_2_)], 1619/1622 [ν(CC)_ph_], 1503/1503 [ν(N4=C5); ν(CC)_ph_; δ(CH)_ph_], 1256/1253 [ν(C10-O13)] and 1172/1173 [δ(CH)_ph_]. Because of the significant overlap between the computed spectra of conformers **1′-a** and **1′-s** (compare Figure 4d,e), unambiguous assignment of individual bands to each conformer is not feasible.

Annealing of cryogenic matrices is a well-established approach for inducing conformational changes in matrix-isolated molecules, thereby enabling the spectroscopic discrimination and characterization of individual conformers [28,29,30,32,33,34,35]. According to the empirical correlation proposed by Barnes [36], interconversion between the **1′-a** and **1′-s** conformers, separated by an energy barrier of 13–14 kJ mol^−1^, would require annealing of the matrix to temperatures of ~45 K. Such temperatures are beyond the operational limit of Ar matrices, as annealing above ~35 K typically results in substantial matrix evaporation and a marked loss of optical transparency. Therefore, the annealing experiments were carried out using Xe as the matrix host, which offers greater thermal stability and permits annealing up to ~65 K without compromising matrix integrity or spectroscopic quality. In the present case, however, annealing of the matrix to 65 K failed to induce any observable spectral changes that could be attributed to changes in the populations of **1′-a** and **1′-s**. This result could indeed be anticipated considering the isoenergetic nature of the two conformers, as predicted by the theoretical calculations.

### 2.3. UV-Induced Photochemistry of Matrix-Isolated ***1***

**Figure 5:**
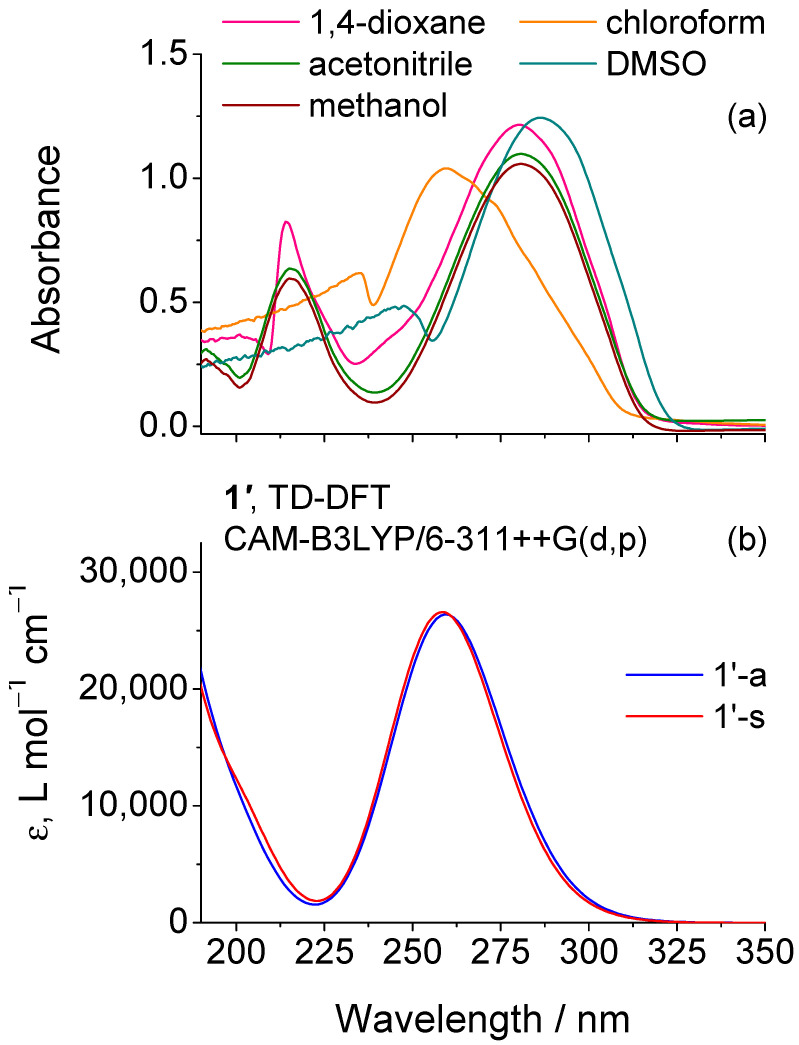
(**a**) UV-Vis absorption spectra of **1** in different solvents compared with the (**b**) UV spectra simulated for the two **1′** conformers (**1′-a** and **1′-s**) from TD-DFT calculations performed at the CAM-B3LYP/6-311++G(d,p) level.

Before conducting the UV irradiation experiments on matrix-isolated **1**, we first measured the UV absorption spectra of the compound in various solvents (Figure 5) to define the optimal excitation window. The maximum of absorption appears at 261 nm in chloroform and undergoes a pronounced bathochromic shift to 281–286 nm in more polar solvents, including 1,4-dioxane, acetonitrile, methanol, and DMSO, reflecting excited state relative stabilization by solvent interactions. The gas-phase UV spectra simulated at the CAM-B3LYP/6-311++G(d,p) level shows excellent agreement with the experimental UV spectrum of the compound in chloroform, predicting a main S_1_←S_0_ ππ* transition at 260 and 259 nm for the **1′-a** and **1′-s** conformers, corresponding to a LUMO ← HOMO promotion (see Tables S2 and S3 for details).

To investigate the photochemical behavior of matrix-isolated **1**, in situ UV-irradiations were carried out using a Xe/Hg lamp. After each irradiation step, an IR spectrum was recorded to monitor potential photochemical transformations. Initial experiments employed a cutoff filter transmitting only wavelengths above 283 nm, which is close to the long-wavelength edge of the compound absorption band in chloroform. However, no significant spectral changes were observed, indicating negligible photoreactivity under these irradiation conditions. Consequently, subsequent irradiations were carried out using unfiltered UV light emitted directly through the cryostat’s outer quartz window (λ > 200 nm), thereby encompassing the entire absorption band profile and enabling excitation of all six lowest singlet states predicted computationally (S_1_–S_6_, 204–260 nm, see Tables S2 and S3). The sequence of irradiations began with an initial exposure of 1.5 min, followed by progressively longer intervals, with the longest reaching a total duration of 100 min. The resulting sequence of IR spectra revealed a gradual decrease in the intensity of the absorption bands associated with **1**, accompanied by the emergence of new bands, indicating the progressive photoconversion of the reactant into new species. After the initial 1.5-min exposure, 16% of **1** was consumed, and a total of 30% was consumed after the complete sequence of irradiations, corresponding to a total exposure time of 250 min. The observed spectral changes are most prominent in the 2300–1700 cm^−1^ region, where **1** does not absorb, see Table 2. Interpretation of the spectral changes induced by the UV irradiations was supported by comparison with the computed IR spectra of all plausible photoproducts, as well as by reference to previously reported spectroscopic signatures of the emerging species.

**Scheme 2:**
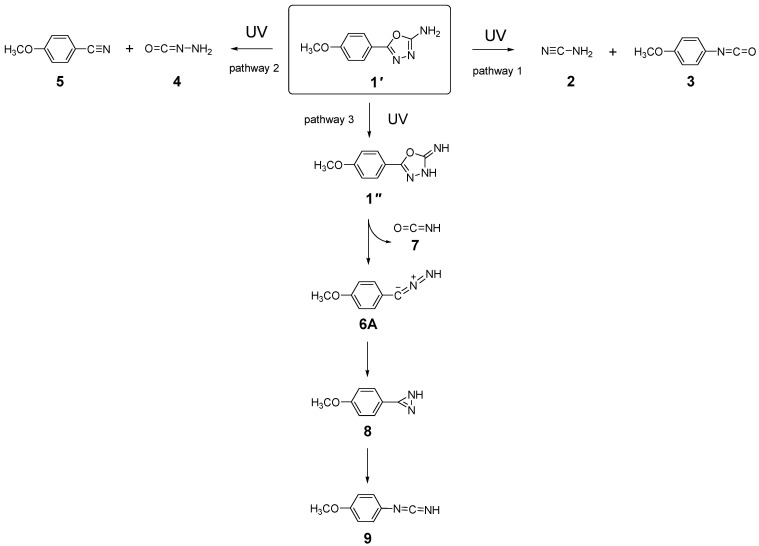
Summary of the photochemical transformations observed upon broadband UV irradiation (λ > 200 nm) of matrix-isolated tautomer **1′**. Pathway 1: Formation of cyanamide **2** and 1-isocyanato-4-methoxybenzene **3** via simultaneous cleavage of the N−N and C2−O1 bonds. Pathway 2: Formation of aminoisocyanate **4** and 4-methoxybenzonitrile **5** through concurrent cleavage of the N−N and C5−O1 bonds. Pathway 3: Generation of 5-(4-methoxyphenyl)-1,3,4-oxadiazol-2(3H)-imine **1″**, followed by subsequent cleavage of the C2−N3 and C5−O1 bonds to yield C-(4-methoxyphenyl)-nitrilimine **6A** and isocyanic acid **7**; species **6A** then undergoes photorearrangement to N-(4-methoxyphenyl)methanediimine **9** via the diazirine intermediate 3-(4-methoxyphenyl)-1*H*-diazirine **8**.

**Figure 6:**
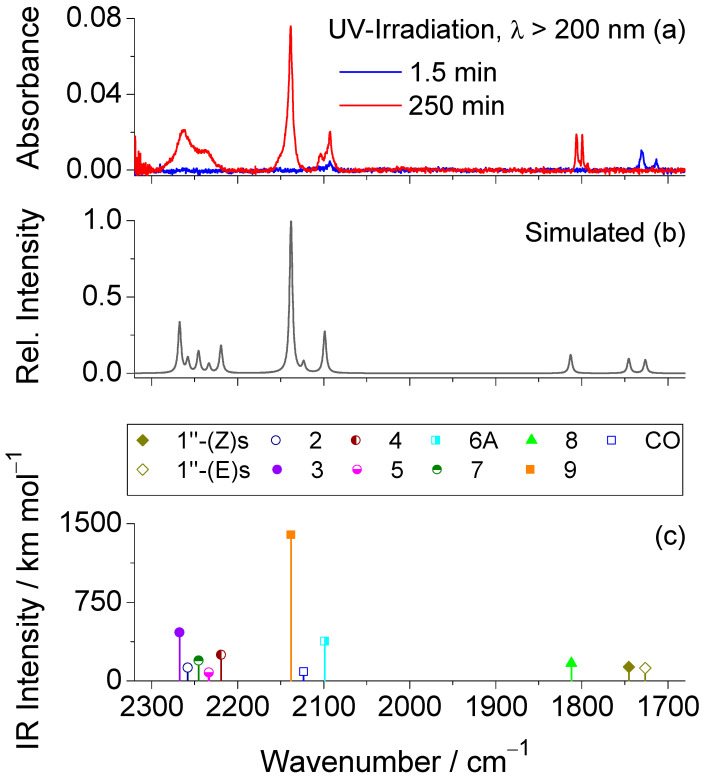
(**a**) 2300−1680 cm^−1^ region of the experimental difference IR spectra obtained by subtracting the spectrum of the as-deposited Ar matrix at 15 K from those recorded after 1.5 min (blue trace) and a total irradiation time of 250 min (red trace) of broadband UV irradiation (λ > 200 nm). (**b**) Simulated composite IR spectrum of the photoproducts, based on harmonic vibrational data computed at the B3LYP/6-311++G(d,p) level, as detailed in the computational methods section. To improve agreement with the experimental spectrum, the intensities of species **3**, **4**, and **7** were scaled by a factor of 0.25, and those of species **1″**(**Z**)**s** and **1″**(**E**)**s** by 0.15. For the remaining photoproducts, the intensities were not scaled. (**c**) Scaled wavenumbers and corresponding IR intensities (scaled as referred above) for all photoproducts, calculated at the same level of theory. For the photoproducts exhibiting two conformers [**1″**(**Z**)**s** and **1″**(**E**)**s**, **3, 6A**, **8** and **9**], only the most stable was considered.

Based on the reported photochemistry of unsubstituted 1,3,4-oxadiazole in an Ar matrix [26] and the established reactivity patterns of related five-membered heterocycles [27,37,38], UV excitation of **1** is expected to induce ring-opening by photocleavage of the formally single N−N and C−O bonds. Cleavage of the N−N and C2−O1 bonds initiates pathway 1, leading to the formation of cyanamide **2** and 1-isocyanato-4-methoxybenzene **3**. Alternatively, cleavage of the N−N and C5−O1 bonds follows pathway 2, resulting in aminoisocyanate **4** and 4-methoxybenzonitrile **5**, as illustrated in Scheme 2. Among these photoproducts, **3** and **4** contain an isocyanate (–N=C=O) group. Their computed IR spectra predict very intense antisymmetric stretches of this group, ν_as_(N=C=O), at 2268 cm^−1^ (Ath = 1850 km mol^−1^) and 2220 cm^−1^ (Ath = 1001 km mol^−1^), respectively (see Table S1). Experimentally, the spectrum recorded after a total irradiation time of 250 min (Figure 6), displays a broad band centered at 2263 cm^−1^ with a shoulder at 2238 cm^−1^, in excellent agreement with the theoretical predictions, as well as with previous experimental data reported for structurally related isocyanates [39,40,41]. Photoproducts **2** and **5** also exhibit absorptions in this region, though their characteristic C≡N stretching vibrations predicted at 2258 and 2233 cm^−1^ are much weaker (Ath = 126 and 80 km mol^−1^, respectively, see Table S3).

In addition to the broad emerging band mentioned above, other new bands are observed at 2138, 2092, and 1806/1799 cm^−1^, which cannot be accounted for by any of the photoproducts formed via pathways 1 or 2. These observations point to the involvement of an alternative photochemical route (pathway 3, see Scheme 2). Notably, a distinct doublet at 1731/1713 cm^−1^ emerges during the initial stages of irradiation and vanishes upon prolonged exposure, indicating its assignment to a primary photogenerated intermediate formed at the onset of the phototransformation. As shown in Figure 6, this doublet band is very well reproduced by the ν(C=N) absorptions predicted for **1**″ (**Z**)**s** and **1″**(**E**)**s** species at 1745 cm^−1^ (Ath = 887 km mol^−1^) and 1726 cm^−1^ (Ath = 813 km mol^−1^), strongly supporting the occurrence of **1′ → 1″** phototautomerization.

Species **1″** exhibits strong UV absorption (see Figure S2 and Tables S4 and S5) and, once formed, may undergo ring opening through OCNH extrusion (pathway 3), similar to the mechanism previously reported by us for a structurally related pyridyl-substituted 1,3,4-oxadiazole-thione in an Ar matrix [27]. This pathway leads to the formation of C-(4-methoxyphenyl)-nitrilimine **6** and isocyanic acid **7**. Identification of **7** is complicated because its predicted strongest ν_as_(N=C=O) band at 2246 cm^−1^ (Ath = 783 km mol^−1^) falls in a congested region where species **2**, **3**, **4** and **5** also absorb. In contrast, species **6** can be confidently assigned based on the absorption band at 2092 cm^−1^, which matches the predicted ν_as_(CCN) vibration at 2080 cm^−1^ (Ath = 385 km mol^−1^), and is consistent with an allenic-type structure [42,43,44,45], here denoted as **6A**. Indeed, *C*-phenyl nitrilimine and phenyl-substituted analogues with allenic character have been photogenerated in an Ar matrix from phenyl tetrazole and corresponding phenyl-substituted tetrazoles [37,38,45,46,47]. In all cases, the characteristic ν_as_(CCN) absorptions were identified in the 2098−2032 cm^−1^ range (in Ar matrices). The close match with the observed band at 2092 cm^−1^ in the present study reinforces the assignment of this new band to **6A**. It is worth noting that nitrilimines can adopt either allenic or propargylic (bond-shift) isomeric forms [37,38,45,46,47]. Computational analysis of the potential energy surface around the CCN bond angle (see Figure S3) reveals a single minimum, characterized by a bent CCN fragment (bond angle ~145°) and a non-planar geometry (CCNH dihedral ~99°), confirming the allenic structure indicated by the spectroscopic results. In contrast, the propargylic isomer (**6P**), characterized by a linear C−C≡N bond angle (~180°) and a planar geometry (C−C≡N−H dihedral ~180°), does not represent a minimum on the potential energy surface and is therefore not photogenerated.

Nitrilimines, once formed from their precursors, typically undergo photorearrangement to their carbodiimide isomeric forms via diazirine intermediates [37,38,45,48,49]. In the present study, the formation of both 3-(4-methoxyphenyl)-1*H*-diazirine **8** and *N*-(4-methoxyphenyl)methanediimine **9** in the photolyzed matrix is supported by the emerging bands at 1806/1799 cm^−1^ and 2138 cm^−1^, respectively. The pair of bands at 1806/1799 cm^−1^ is characteristic of the ν(C=N) stretching vibration of the strained three-membered diazirine ring in species **8**, and matches well with the computed value at 1776 cm^−1^. These bands are also consistent with literature reports for structurally related diazirines in Ar matrices, such as 3-phenyl-1*H*-diazirine (1789 cm^−1^) [47], and the corresponding 4-amino- and 4-hydroxyphenyl-substituted analogues (1785 and 1781 cm^−1^, respectively) [37,38], confirming the identity of the species formed. The strong band observed at 2138 cm^−1^ is assigned to the antisymmetric cumulenic stretching vibration ν_as_(N=C=N) of carbodiimide **9**, theoretically predicted at 2139 cm^−1^ (Ath = 1368 km mol^−1^). A band at this position and ascribed to the same species was also reported during the photofragmentation of 4-(4-pyridyl)-1,3,4-oxadiazole-2(3*H*)-thione [27], a structurally analogous compound to tautomer **1″**. This assignment is further supported by literature data for related carbodiimides, which consistently exhibit characteristic ν_as_(N=C=N) features in this spectral region [37,38,45,47]. These findings corroborate the formation of species **9** as a photorearrangement product of the nitrilimine intermediate. However, it should be noted that this band may also contain minor contributions from carbon monoxide (CO), possibly arising from side reactions involving minor photodecomposition processes.

**Figure 7:**
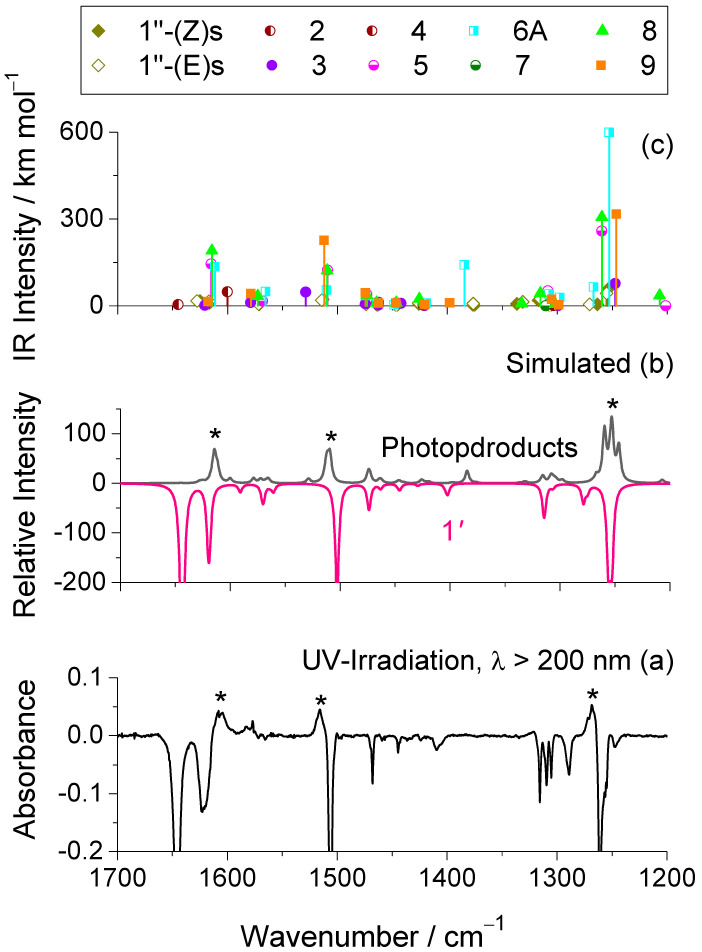
(**a**) 1700−1200 cm^−1^ region of the experimental difference IR spectra obtained by subtracting the spectrum of the as-deposited Ar matrix at 15 K from those recorded after 250 min of broadband UV irradiation (*λ* > 200 nm). (**b**) Simulated composite IR spectrum of the photoproducts (positive spectrum) and reactant **1′** (negative spectrum), based on harmonic vibrational data computed at the B3LYP/6-311++G(d,p) level, as detailed in the computational methods section. The emerging bands are marked with an asterisk. The most intense bands in the simulated positive spectrum, corresponding to the emerging features in (**a**), are marked with an asterisk. To improve agreement with the experimental spectrum, the intensities of species **3**, **4**, and **7** were scaled by a factor of 0.25, and those of species **1″**(**Z**)**s** and **1″**(**E**)**s** by 0.15. For the remaining photoproducts, the intensities were not scaled. For the photoproducts exhibiting two conformers [**3**, **6A**, **8**, **9**, **1″**(**Z**)**s** and **1″**(**E**)**s**], only the most stable one was used in the simulation. (**c**) Scaled wavenumbers and corresponding IR intensities (scales as referred above) for all photoproducts, calculated at the same level of theory. As in (**b**), only the most stable conformer was considered for species **3**, **6A**, **8**, **9**, **1″**(**Z**)**s** and **1″**(**E**)**s**.

The spectral evolution in the region below 1700 cm^−1^ following UV irradiation is presented in Figure 7a. Interpretation of this region is very challenging due to significant overlap between bands of the reactant and those of the photoproducts, as well as the close proximity of the predicted vibrational modes of the latter. Nevertheless, the composite simulated IR spectrum, which was constructed by summing the calculated spectra of all proposed photoproducts (Figure 7b), shows good agreement with the experimentally observed difference spectra. Three prominent new bands emerge at 1607, 1516, and 1269 cm^−1^, which correlate well with the computed frequencies at 1614, 1511, and 1253 cm^−1^, respectively. These features originate primarily from species **5**, **6**, **8**, and **9**. The band at 1607 cm^−1^ is mainly attributed to aromatic C=C stretching vibrations [ν(C=C)_ph_], while that at 1516 cm^−1^ arises from a combination of aromatic C=C stretching and in-plane C–H bending modes [ν(C=C)_ph_; δ(CH)_ph_]. The feature at 1269 cm^−1^ is assigned to the C2–O1 stretching vibration [ν(C–O)], which, in the case of species **6**, has a significant contribution from the N−H in-plane bending mode [δ(NH)], as predicted at 1253 cm^−1^.

Overall, the consistency between observed and computed band positions strongly supports the proposed photochemical pathways, confirming the formation of the identified photoproducts and reinforcing the mechanistic interpretation of the UV-induced transformations occurring in the matrix.

## 3. Methods

### 3.1. Experimental Methods

For the matrix-isolation experiments, commercially available 2-amino-5-(4-methoxyphenyl)-1,3,4-oxadiazole **1** (solid, m.p. 241–249 °C) was obtained from Sigma-Aldrich (purity > 97%, Saint Louis, MO, USA) and used without further purification. High-purity Ar and Xe (99.99%) were used as the host gases. A small quantity of **1** was placed inside a miniature glass oven which was then attached to the vacuum chamber of a closed-cycle helium cryostat with a DE-202 A expander (APD cryogenics, Allentown, PA, USA). The pressure inside the vacuum chamber goes down to ~3.5 × 10^−5^ mbar. Before cooling the cryostat, a freeze-pump-thaw procedure was performed to purify the sample from volatile impurities. To prepare the low-temperature matrices, the solid compound was sublimated by applying potential and current with a Vitecom DC Power Supply 75-HY5003 model power meter device (Vitec POWER GmbH, Ganserndorf, Austria). The highest current and potential values applied were approximately 0.60 A and 2.3 V, respectively. Vapors from the sublimation of **1** were co-deposited with a large excess of Ar or Xe onto a CsI optical substrate, cooled down to 15 K. The temperature of the CsI window was measured directly at the sample holder by using a silicon diode sensor connected to a digital controller (model 9650-1, Scientific Instruments, Inc., West Palm Beach, United States), ensuring the stabilization of temperature with an accuracy of 0.1 K.

Mid-IR spectra (4000–400 cm^−1^) of the matrix-isolated compound were acquired on a Thermo Nicolet 6700 FTIR spectrometer (Thermo Fisher Scientific, Waltham, MA, USA) at a resolution of 0.5 cm^−1^, using a deuterated triglycine sulfate (DTGS) detector and a Ge/KBr beam splitter. To prevent interference from atmospheric H_2_O and CO_2_, the system was continuously purged with dry, CO_2_-filtered air.

Broadband UV irradiations (*λ* > 200, 283 nm) were carried out for the compound isolated in solid Ar. The irradiations were performed directly through the outer quartz window of the cryostat using a 500 W Hg(Xe) arc lamp (Oriel Instruments, Newport, RI, USA) with the output power set to 200 W. In all irradiation experiments, a water filter was used to prevent heating of the matrix.

UV-Vis spectra of **1** in various spectroscopic-grade solvents (Sigma-Aldrich) were recorded from 800 to 190 to nm a PerkinElmer Lambda 35 UV–Vis spectrophotometer (PerkinElmer, Inc., Shelton, CT, USA). All measurements were performed at room temperature in a quartz cuvette (1 cm path length).

### 3.2. Computational Methods

All Quantum mechanical computations were carried out using Gaussian 09 (revision A.02) [50] and Gaussian 16 (Revision B.01) [51] suite of programs. The geometries of the reactants and photoproducts were fully optimized using the DFT(B3LYP) functional [52,53,54] and the 6-311++G(d,p) basis set, employing “tight” convergence criteria. For the title molecule **1**, additional optimizations were carried out at the B3LYP/aug-cc-pVTZ and MP2 [55]/6-311++G(d,p) levels. Subsequently to the geometry optimizations, harmonic vibrational calculations were performed at the respective levels. The Cartesian coordinates of the optimized geometries of all species investigated are available in the Supplementary Material. The B3LYP/6-311++G(d,p) vibrational data was used to support the interpretation of the matrix-isolation IR spectra. To address limitations arising from basis set deficiencies, vibrational anharmonicity, incomplete electron correlation treatment and matrix effects [56], the calculated wavenumbers were scaled using factors of 0.960 for the region of 4000–1800 cm^−1^ and 0.980 in the range of 1800–400 cm^−1^ [37,38,57,58,59]. For graphical comparison with the experimental spectra, the wavenumbers and IR intensities (in km mol^−1^) extracted from the vibrational calculations were convoluted with the Lorentzian functions with a full-width-at-half-maximum (FWHM) of 2 or 4 cm^−1^ and peak heights matching the calculated (unscaled) IR intensities. The analysis was performed with the “VibAnalysis” software [60] supported by animation of the computed vibrations using the Chemcraft program (version 1.8) [61]. Theoretical UV absorption spectra for all species relevant to this study were obtained from TD-DFT [62] calculations at the CAM-B3LYP [63]/6-311++G(d,p) level, which is known to provide improved accuracy over the standard B3LYP functional for predicting vertical excitation energies across a wide range of systems, including heterocyclic compounds [64,65,66]. Each electronic transition was convoluted with a Lorentzian function (full width at half maximum, FWHM = 0.33 eV) to simulate the overall spectral profile.

## 4. Conclusions

In this study, we have conducted a comprehensive investigation of the molecular structure, spectroscopic properties, and UV-induced photochemistry of 2-amino-5-(4-methoxyphenyl)-1,3,4-oxadiazole **1** under cryogenic matrix-isolation conditions. Quantum chemical calculations revealed that the two conformers of the amino tautomer (**1′-a** and **1′-s**) are the predominant species in the gas phase. The B3LYP and MP2 computed energy difference between them was found to be less than 0.5 kJmol^−1^, indicating that both conformers are nearly isoenergetic. Matrix-isolated IR spectra confirmed exclusive trapping of these conformers, in excellent agreement with the theoretical predictions. Annealing experiments failed to induce detectable conformational interconversion, confirming the isoenergetic character of the two experimentally relevant conformers of the studied molecule.

Upon broadband UV irradiation (*λ* > 200 nm), compound **1** underwent efficient phototransformation through three distinct reaction pathways. Pathways 1 and 2 involved classical N–N and C–O bond cleavages of the oxadiazole ring, yielding isocyanate derivatives as major photoproducts, with their characteristic features identified in the 2265–2230 cm^−1^ region. A third pathway was also identified, initiated by a phototautomerization from the amino to the imino form **1″**, evidenced by the appearance of a characteristic doublet at 1731/1713 cm^−1^ in the early stages of irradiation, followed by a ring-opening process that led to the formation of a nitrilimine intermediate, confidently assigned based on the absorption band at 2092 cm^−1^. This reactive species has been found possess a dominant allenic character (calculations demonstrated that the putative propargylic bond-shift isomer of this species does not correspond to a minimum energy structure) and subsequently rearranged into its isomeric carbodiimide via a diazirine-mediated mechanism. Both species were identified by their characteristic infrared bands at 1806/1799 cm^−1^ and 2138 cm^−1^, respectively.

All major photoproducts were identified through their characteristic IR signatures, supported by quantum chemical calculations and literature benchmarks. The results highlight the complex and multichannel nature of the photochemistry of functionalized 1,3,4-oxadiazoles and demonstrate how subtle substituent effects can modulate tautomeric equilibria and photochemical reaction pathways. The insights gained here expand our fundamental understanding of the structure–reactivity relationships in this important class of heterocycles and may inform future applications in materials science and photopharmacology.

## Figures and Tables

**Table 1:** Relative electronic energies (Δ*E*_el_), zero-point corrected energies (Δ*E*_ZPVE_), Gibbs energies, Boltzmann populations and dipole moments computed for the different isomers of **1** at the different levels of theory *^a^*.

Isomer	Δ*E*_el_	Δ*E*_ZPVE_	ΔG^0^	Pop (%)	μ (Debye)
B3LYP/6-311++G(d,p)					
**1′a**	0.0	0.0	0.0	53.44	3.13
**1′s**	0.2	0.2	0.4	46.22	5.39
**1″-**(**Z**)**s**	17.4	16.0	14.0	0.15	4.30
**1″-**(**Z**)**a**	17.8	16.4	14.5	0.19	3.94
**1″-**(**E**)**s**	27.7	27.0	26.9	0.00	2.34
**1″-**(**E**)**a**	28.5	27.7	27.4	0.00	4.36
B3LYP/aug-cc-pVTZ					
**1′-a**	0.0	0.0	0.0	52.5	3.17
**1′-s**	0.1	0.2	0.3	47.5	5.25
MP2/6-311++G(d,p)					
**1′-a**	0.0	0.4	0.5	45.5	3.28
**1′-s**	0.2	0.0	0.0	54.5	5.45

*^a^* Energies are given in kJ mol^−1^. Gibbs free energies and Boltzmann populations (estimated from the Δ*G*^0^ values) are reported at 298.15 K. See Figure 2 for the geometric representation of the isomers.

**Table 2:** Experimental IR spectra (Ar and Xe matrices, 15 K) compared with the B3LYP/6-311++G(d,p) vibrational frequencies (ν, cm^−1^) and infrared intensities (Ath, km mol^−1^) calculated for the **1′-a** and **1′-s** conformers forms of 2-amino-5-(4-methoxyphenyl)-1,3,4-oxadiazole **1** and approximate vibrational assignments *^a^*.

Exp. (Ar, 15 K) *^a^*	Exp. (Xe, 15K) *^a^*	Calc. 1′-a		Calc. 1′-s		Approximate Assignments *^c^*
ν_Exp_ *^b^*	ν_Exp_ *^b^*	ν_Calc._	Ath	ν_Calc._	Ath	
3521	3507	3533	52.1	3532	51.9	ν(NH_2_)_as_
3426	3411	3432	68.1	3432	66.9	ν(NH_2_)_s_
1645	1642	1644	454.0	1644	435.0	ν(C2=N3) − ν(C2=C14)
1624/1620	1619	1619	158.9	1620	182.7	ν(CC)_ph_
1593/1590	1596/1587	1591	27.2	1591	5.1	ν(C5=N4); ν(CC)_ph_; δ(NH_2_)
1572	1569	1570	54.6	1571	28.4	ν(C2=N3); δ(NH_2_)
1566	1563	1564	7.7	1561	30.5	ν(C5=N4) − ν(C5−C6) + ν(C2=N3); ν(CC)_ph_
1505	1504	1503	214.4	1503	233.1	δ(CH)_ph_; ν(C5=N4); ν(C9−O12); ν(CC)_ph_
1468	1463	1474	60.8	1474	44.1	δ(CH_3_)_as_
1460	1455	1463	9.6	1463	9.6	δ(CH_3_)_as′_
1445/1443	1441	1447	14.5	1446	14.3	δ(CH_3_)_s_
1424	1420	1424	0.8	1429	8.7	ν(C2−O1) + ν(C5−O1); ν(CC)_ph_; δ(CH)_ph_
1410/1404 (sh)	1408	1404	12.2	1402	40.6	ν(C2−O1) + ν(C5−O1) − ν(C2−N3) − ν(C2−N14)
1316/1310	1315/1311	1314	96.7	1315	43.6	ν(CC)_ph_
1305	1307	1307	3.7	1306	11.2	δ(CH) _ph_
1289	1289	1275	27.9	1279	73.5	ν(C5-O1) − ν(C5−C6) + ν(C5−N4); δ(CH)_ph_
1261/1256 (sh)	1258	1256	328.8	1253	289.8	ν(C9−O12)
1185	1183	1178	7.2	1178	11.7	*ρ*(CH_3_)
1175	1174	1172	86.8	1173	77.4	δ(CH)_Ph_
n.o.	n.o.	1144	0.7	1144	0.6	*ρ*(CH_3_)′
1116	1115	1116	2.2	1116	8.2	ν(C2=N3) − ν(C2−N14); *ρ*(NH_2_); δ(CH)_ph_
1114	1112	1113	10.2	1113	4.4	δ(CH)_ph_; *ρ*(NH_2_)
1068	1066	1054	4.5	1053	37.3	ν(C5−O1) − ν(C13−O12)
1045/1038	1042/1035	1035	54.9	1033	16.4	ν(C13−O12) − ν(N3−N4)
1026/1016/1011	1026/1016/1009	1020	84.1	1021	89.5	ν(N3−N4) − ν(C2−O1)
1000	1000	1001	11.2	1001	13.0	δ(CCC)_ph_
967	965	969	4.6	969	2.4	ν(C2−O1) + ν(N3−N4)
n.o.	n.o.	959	0.2	968	< 0.1	γ(C)_ph_; γ(CH)_ph_
951	950	945	16.1	945	19.9	δ(C5N4N3); ν(C5-O1)
n.o.	n.o.	943	0.4	930	0.5	γ(C)_ph_; γ(CH)_ph_
839/835	836/833	832	51.7	837	44.8	γ(C)_ph_; γ(CH)_ph_
809	808	808	1.8	803	8.5	γ(C)_ph_; γ(CH)_ph_
802	800	798	5.6	798	4.1	δ(CCC)_ph_; ν(CC)_ph_; δ(C9-O12)
739	737	743	35.7	743	38.9	γ(C)_ox_
n.o.	n.o.	731	3.2	731	3.9	δ(O1-C2-N3); ν(C2-N14)
721	720	710	11.7	710	11.8	γ(C)_ph_; γ(C)_ox_
686	684	670	1.5	670	1.3	γ(N)_ox_; γ(C)_ox_
637/635	637/635	635	1.8	637	4.9	δ(CCC)_ph_
611	611	605	30.3	607	24.9	δ(CCC)_ph_; ν(C9−O12)

*^a^* Because of their minor relevance for the present study, absorptions bands falling in the 3100–2800 cm^−1^ region, which are assigned to the CH and CH_3_ stretching vibrations, have not been included in this table. *^b^* Calculated harmonic wavenumbers (in cm^−1^) were multiplied by 0.960 (above 1800 cm^−1^) or 0.980 (below 1800 cm^−1^).*^c^* Abbreviations: ν = stretching, δ = in-plane bending, γ = out-of-plane bending, s = symmetric, and as = antisymmetric; ph = phenyl ring; ox = 1,3,4-oxadiazole ring.

## Data Availability

Data are contained within the article and Supplementary Materials.

## Supplementary Materials

The following supporting information can be downloaded at: https://www.mdpi.com/article/10.3390/molecules30163444/s1, Section S1: Figures. Figure S1, Experimental IR spectra of 2-Amino-5-(4-methoxyphenyl)-1,3,4-oxadiazole **1** isolated in Ar and Xe matrices (15 K), compared with the spectra calculated for the isomeric forms of tautomer 5-(4-methoxyphenyl)-1,3,4-oxadiazol-2(3*H*)-imine **1″**; Figure S2, TD-DFT UV absorption spectra simulated at the CAM-B3LYP/6-311++G(d,p) level for the species generated upon broadband UV irradiation (*λ* > 200 nm) of **1**; Figure S3, B3LYP/6-311++G(d,p) relaxed potential energy scans as a function of the CCN angle for 4-methoxyphenyl-nitrilimine **6**. Section S2: Tables. Table S1, B3LYP/6-311++G(d,p) calculated vibrational frequencies (ν/cm−1; scaled) and infrared intensities (Ath/km mol^−1^; unscaled) for the species resulting from the UV-induced photochemistry of **1**; Tables S2–S5, Data extracted from the TD-DFT calculations performed for **1** and **1″** at the CAM-B3LYP/6-311++G(d,p) level. Section S3: Computational data. Cartesian coordinates of the fully optimized structures for the species relevant to this work.
