# Supplementary material for: Molecular Structure, Matrix-Isolation IR Spectrum and UV-Induced Transformations of 2-Amino-5-(4-Methoxyphenyl)-1,3,4-Oxadiazole
Source: Molecules. 2025 Aug 21;30(16):3444. doi: 10.3390/molecules30163444 (PMC12388349; doi:10.3390/molecules30163444)
Supplement: Supplementary file 1 [file molecules-30-03444-s001.zip › molecules-3807324-supplementary.pdf]

Article

Molecular Structure, Matrix-Isolation IR Spectrum and UV-Induced Transformations of 2-Amino-5-(4-Methoxyphenyl)-1,3,4-Oxadiazole

İsa Sidir [1,2,*], Susy Lopes [2], Rui Fausto [2,3] and A. J. Lopes Jesus [4,*]

[1] Department of Physics, Faculty of Sciences and Letters, Bitlis Eren University, Bitlis 13000, Türkiye
[2] University of Coimbra, CQC–IMS, Department of Chemistry, 3004–535 Coimbra, Portugal; susylopes@qui.uc.pt (S.L.); rfausto@ci.uc.pt (R.F.)
[3] Spectroscopy@IKU, Faculty of Sciences and Letters, Department of Physics, Istanbul Kultur University, Ataköy Campus, Bakırköy 34156, Istanbul, Türkiye
[4] University of Coimbra, CQC-IMS, Faculty of Pharmacy, 3000-548 Coimbra, Portugal.
* Correspondence: isidir@beu.edu.tr (I.S.); ajorge@ff.uc.pt (A.J.L.J.)

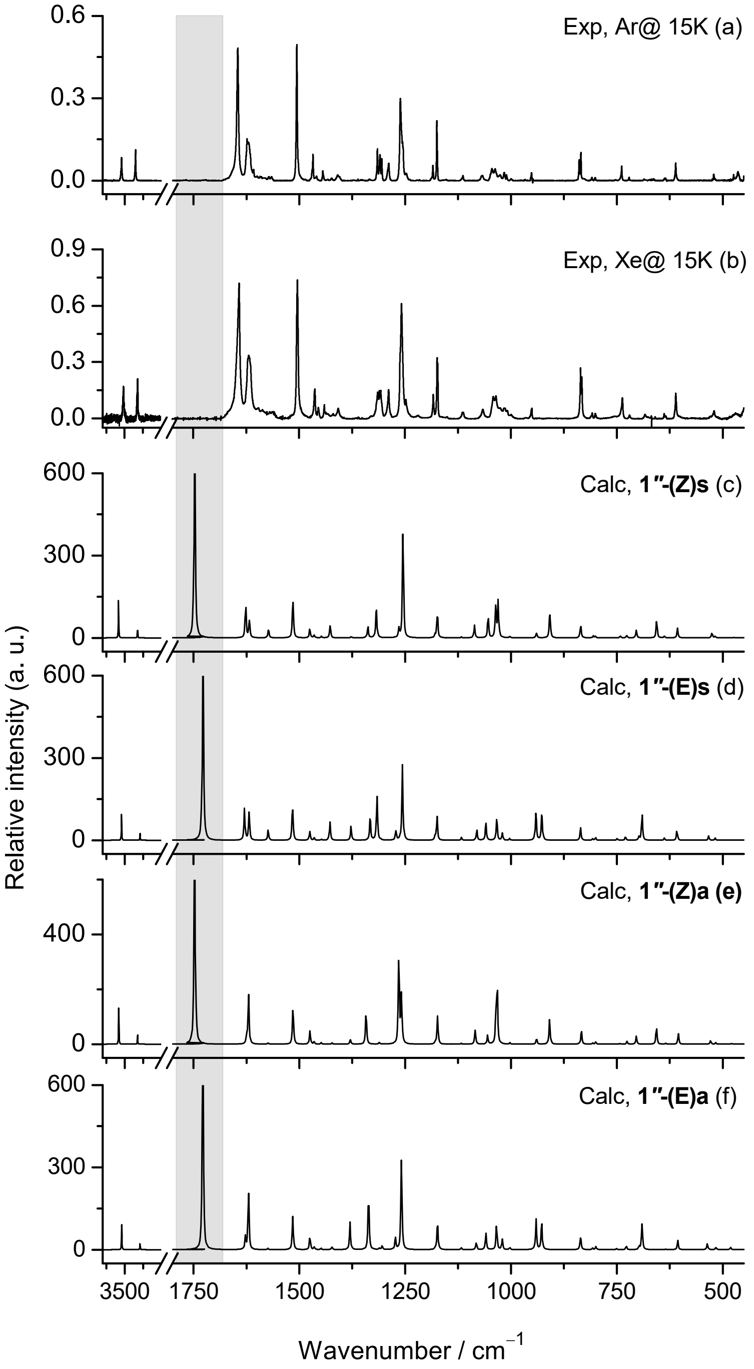

Figure S1. (a,b) Experimental IR spectra of of 2-Amino-5-(4-methoxyphenyl)-1,3,4-oxadiazole **1** isolated in Ar and Xe matrices (15 K), compared with the (c-f) spectra calculated for the four isomeric forms adopted by tautomer 5-(4-methoxyphenyl)-1,3,4-oxadiazol-2(3H)-imine **1″**, simulated from the wavenumbers (scaled) and IR intensities extracted from the vibrational calculations carried out at the B3LYP/6-311++G(d,p) level of theory. The grey rectangle highlights the region where this tautomeric form exhibits its strongest absorption at 1745-1726 cm^{-1}, assigned to the stretching vibration of the exocyclic C=N bond [ν(C=N)], which is absent in the experimental spectrum.

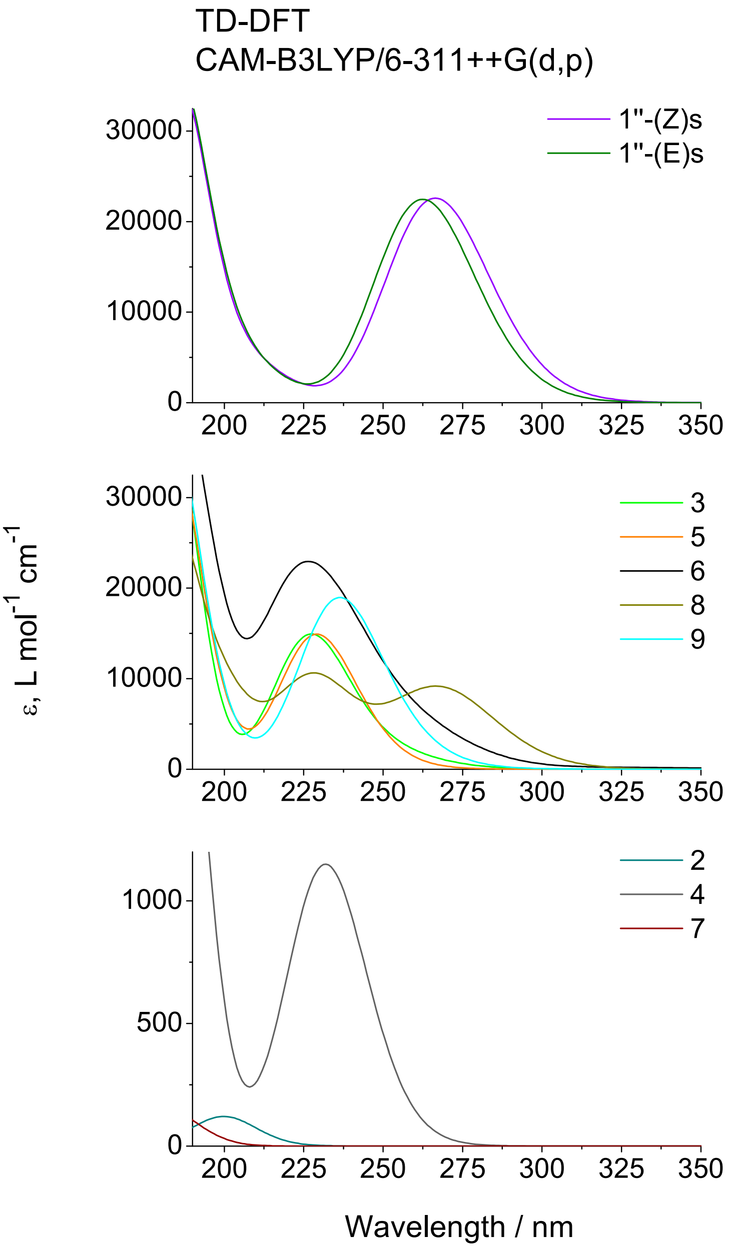

Figure S2. TD-DFT UV absorption spectra simulated at the CAM-B3LYP/6-311++G(d,p) level for the species generated upon broadband UV irradiation (λ > 200 nm) of matrix-isolated 2-amino-5-(4-methoxyphenyl)-1,3,4-oxadiazole **1**.

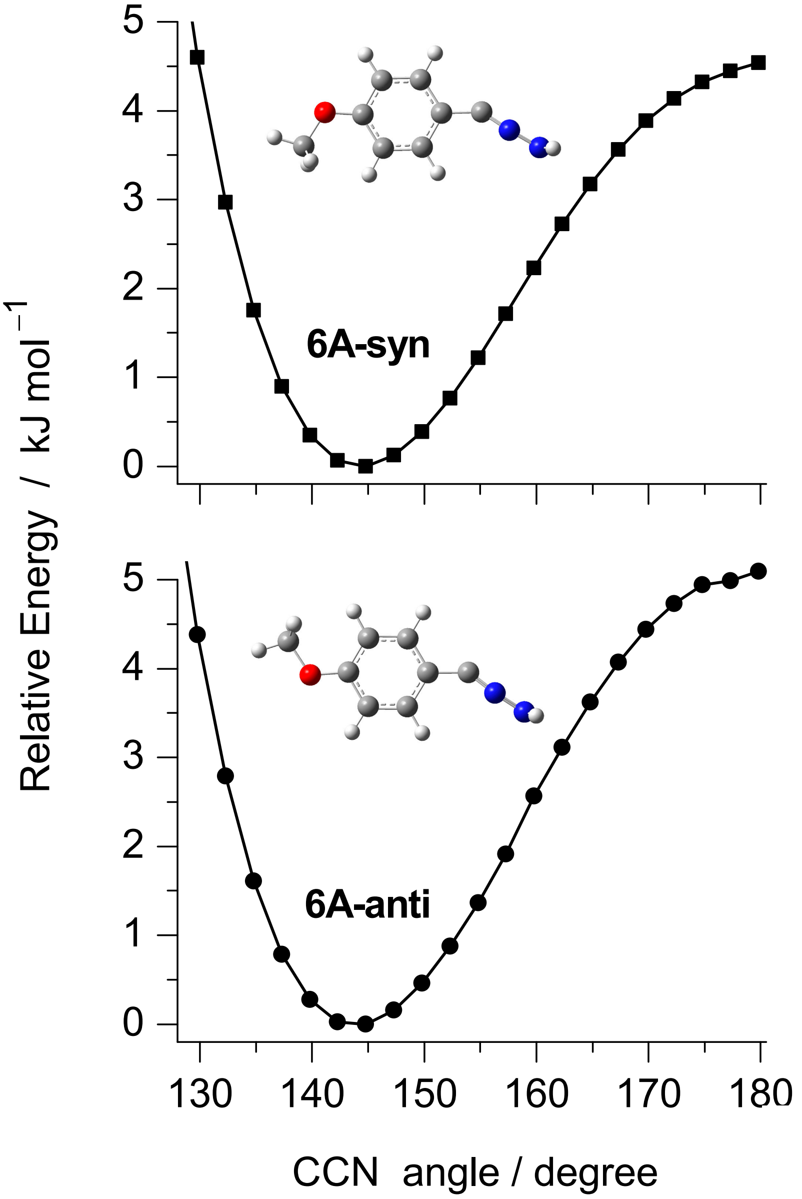

Figure S3. B3LYP/6-311++G(d,p) relaxed potential energy scans as a function of the CCN angle for C-(4-methoxyphenyl)-nitrilimine **6**, considering both syn (top) and anti (bottom) orientations of the methoxy (OCH_3) and nitrilimine (CNN) groups. The CCN angle was incrementally constrained in 2.5° steps, while all other geometrical parameters were fully optimized at each point. For both scans, only one minimum was identified, characterized by bent CCN fragment (CCN angle ~145º) and a non-planar geometry (CCNH dihedral ~ 99º), consistent with an allenic-type structure, labeled as **6A**. The propargylic isomer, characterized by a CCN angle of ~180° and planar geometry (CCNH dihedral ~ 180º), does not represent a minimum on the potential energy surface.

2. Tables

Table S1. B3LYP/6-311++G(d,p) calculated vibrational frequencies (ν/cm^{-1}; scaled) and infrared intensities (A^{th}/km mol^{-1}; unscaled) for species resulting from the UV-induced photochemistry of 2-Amino-5-(4-methoxyphenyl)-1,3,4-oxadiazole **1**.[a]

**1″-(Z)s**		**1″-(Z)a**		**1″-(E)s**		**1″-(E)a**		**2**		**3**	
ν	I	ν	I	ν	I	ν	I	ν	I	ν	I
3549	139	3548	135	3529	97	3528	93	3503	71	3081	5
3419	35	3419	36	3402	25	3402	25	3413	43	3068	2
3083	6	3081	7	3083	6	3081	7	**2258**	**126**	3063	6
3074	3	3076	2	3074	3	3077	1	1600	48	3049	7
3066	3	3063	0	3068	3	3063	0	1166	0	3010	20
3060	3	3062	5	3060	2	3062	4	1075	10	2941	37
3013	20	3013	20	3013	20	3013	20	535	117	2884	57
2947	34	2947	34	2948	33	2947	34	469	140	**2268**	**1850**
2888	59	2889	60	2889	59	2888	60	402	0	1621	8
**1745**	**887**	**1746**	**897**	**1726**	**806**	**1726**	**813**			1579	42
1625	115	1623	27	1628	115	1626	47			1529	189
1616	69	1618	186	1617	101	1618	203			1475	28
1571	33	1572	5	1572	40	1573	4			1463	10
1514	146	1513	140	1514	129	1514	121			1462	47
1474	35	1473	49	1474	32	1473	48			1442	35
1463	10	1464	10	1464	10	1464	10			1421	5
1446	5	1447	6	1446	3	1447	5			1305	25
1426	46	1421	5	1426	67	1421	10			1299	5
1376	4	1378	20	1377	52	1379	101			1247	307
1337	45	1341	117	1331	91	1335	203			1178	11
1317	124	1310	7	1315	163	1310	5			1168	34
1306	1	1304	1	1306	2	1303	14			1144	1
1264	32	1264	310	1271	33	1272	46			1123	29
1254	382	1258	178	1255	286	1258	327			1108	14
1178	11	1177	7	1178	13	1178	5			1037	69
1173	94	1172	102	1174	88	1172	106			1003	1
1144	1	1144	1	1144	1	1144	1			941	0
1116	3	1117	3	1116	11	1116	7			921	1
1085	46	1084	52	1080	40	1081	24			825	69
1053	76	1054	32	1058	68	1058	61			813	0
1035	123	1035	83	1033	85	1033	92			796	2
1029	132	1032	217	1019	25	1020	38			709	0
1002	4	1002	5	1002	6	1002	7			693	35
965	0	959	0	965	0	962	0			641	12

939	19	942	1	940	94	943	3			614	31
935	1	939	20	939	14	940	112			558	27
908	101	908	100	926	112	927	107			511	10
835	50	833	53	835	50	835	51			508	5
805	8	806	4	807	6	807	5			420	0
800	5	799	9	800	10	799	11			383	6
741	6	742	2	749	7	750	4			374	1
726	10	725	10	729	13	727	13			364	0
704	28	703	29	697	13	697	12			234	1
681	0	681	0	690	92	690	94			221	7
655	61	656	60	667	1	667	0			159	3
637	6	634	3	637	8	634	1			87	3
606	37	604	38	608	36	606	34			76	1
525	18	528	13	533	16	536	24			42	0
518	6	516	6	517	9	515	9				
473	2	479	3	475	1	481	9				
418	4	414	1	417	1	413	0				
393	36	394	40	386	14	382	18				
379	3	360	10	378	14	365	3				
318	61	319	62	310	74	312	72				
272	1	299	1	274	0	299	6				
237	5	233	2	242	9	242	9				
235	2	221	1	237	1	222	2				
184	3	186	4	205	10	206	8				
109	14	106	17	150	17	151	16				
99	0	100	1	102	1	101	0				
67	11	73	10	98	2	98	1				
50	9	55	6	56	3	59	4				
45	1	39	1	48	3	40	0				

Table S1. Continued.

**4**		**5**		**6A**		**7**		**8**		**9**	
ν	I	ν	I	ν	I	ν	I	ν	I	ν	I
3391	11	3084	4	3258	2	3542	170	3234	5	3429	69
3325	2	3075	2	3082	7	**2246**	**783**	3083	5	3080	6
**2219**	**1001**	3062	1	3069	3	1311	1	3072	2	3066	2
1645	18	3061	3	3060	4	749	200	3056	1	3061	7
1422	12	3016	17	3053	2	620	4	3054	2	3045	5
1303	2	2952	31	3014	19	557	97	3017	17	3009	21
1044	91	2892	50	2946	33			2954	31	2940	38

847	5	**2233**	**80**	2888	59			2893	51	2884	62
640	45	**1615**	**144**	2080	385			**1776**	**169**	**2138**	**1395**
581	30	1568	16	1612	134			**1614**	**191**	1618	14
203	15	**1509**	**124**	1565	49			1573	34	1579	43
44	59	1473	39	1510	54			**1509**	**122**	**1512**	**226**
		1464	10	1474	36			1473	36	1475	45
		1446	10	1464	10			1464	10	1463	9
		1420	4	1448	3			1446	10	1447	9
		1308	52	1419	10			1426	24	1421	4
		1304	18	1385	141			1332	8	1398	10
		**1259**	**258**	1307	41			1316	43	1305	22
		1201	0	1298	29			1302	6	1299	3
		1178	4	1267	65			**1260**	**306**	**1246**	**317**
		1170	65	1253	599			1207	36	1178	11
		1144	1	1179	11			1178	6	1167	37
		1113	8	1171	110			1165	107	1144	1
		1031	64	1144	1			1144	1	1121	13
		1005	0	1109	16			1115	4	1107	13
		959	0	1106	11			1067	30	1038	64
		938	1	1035	67			1029	71	1003	1
		834	58	1003	1			1002	2	943	0
		815	3	958	0			986	35	924	1
		805	6	938	1			963	1	**887**	**462**
		722	0	832	59			939	1	828	69
		682	19	812	5			833	55	818	1
		646	1	804	4			812	5	797	2
		559	0	717	2			807	7	715	1
		557	23	686	10			713	1	715	19
		493	5	637	16			687	6	640	15
		476	1	603	67			634	3	616	43
		411	0	556	73			571	24	570	48
		378	1	501	13			543	40	508	25
		275	0	490	1			482	2	506	2
		253	4	418	2			475	2	433	62
		213	0	370	16			468	8	421	1
		141	4	367	3			411	0	381	1
		123	9	352	9			317	0	378	4
		72	0	312	38			270	2	341	27
				229	5			249	5	233	3
				222	10			212	1	222	5
				159	13			123	2	153	9
				94	5			120	9	92	1
				72	4			88	3	85	3

				44	2			68	1	40	3

[a] Calculated frequencies are scaled by 0.960 in the range of 4000 - 1800 cm^{-1}, and 0.980 below 1800 cm^{-1}. For the photoproduts exhibiting two conformers [**1″(Z)s** and **1″(E)s**, **3, 6A**, **8** and **9**], only the most stable was considered.

Table S2. Excitation energies (λ, in nm), oscillator strengths (*f*), main orbital transitions (with corresponding amplitudes in parentheses) and isosurface representations of the orbitals involved for the six longest singlet states of conformer **1′-a** of 2-Amino-5-(4-methoxyphenyl)-1,3,4-oxadiazole **1**, obtained from TD-DFT calculations at the CAM-B3LYP/6-311++G(d,p) level of theory.

State	*λ*	*f*	Major contribution	Donor orbital	Acceptor orbital
S1	260.15	0.6037	HOMO→ LUMO (0.67)	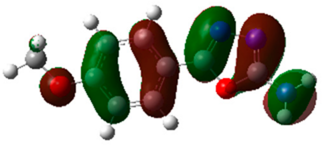 HOMO	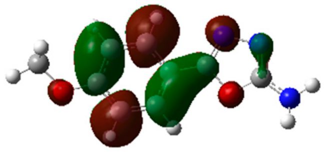 LUMO
S2	251.03	0.0562	HOMO→ LUMO+3 (0.58)	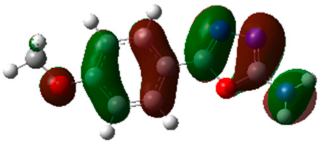 HOMO	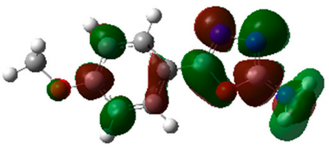 LUMO+3
S3	232.54	0.0043	HOMO→ LUMO+1 (0.63)	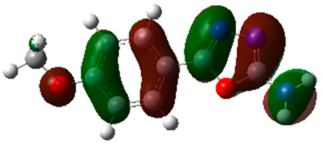 HOMO	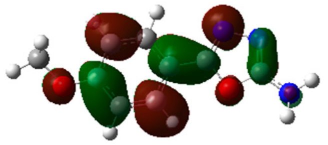 LUMO+1
S4	216.47	0.0028	HOMO→ LUMO+2 (0.60)	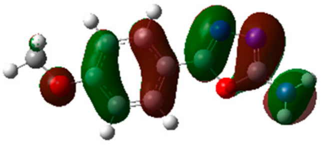 HOMO	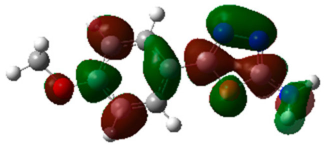 LUMO+2
S5	207.84	0.0029	HOM-3→ LUMO (0.64)	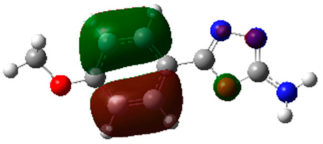 HOMO-3	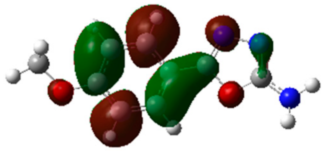 LUMO
S6	202.40	0.0516	HOMO-1→ LUMO (0.45)	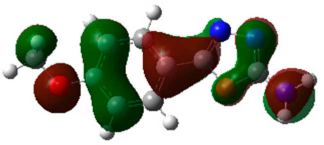 HOMO-1	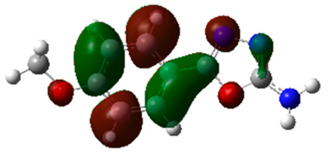 LUMO

Table S3. Excitation energies (λ, in nm), oscillator strengths (*f*), main orbital transitions (with corresponding amplitudes in parentheses) and isosurface representations of the orbitals involved for the six longest singlet states of conformer **1′-s** of 2-Amino-5-(4-methoxyphenyl)-1,3,4-oxadiazole **1**, obtained from TD-DFT calculations at the CAM-B3LYP/6-311++G(d,p) level of theory.

State	*λ*	*f*	Major contribution	Donor orbital	Acceptor orbital
S1	258.79	0.6334	HOMO → LUMO (0.68)	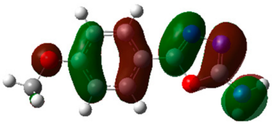 HOMO	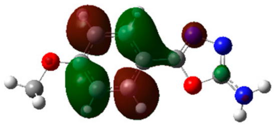 LUMO
S2	250.63	0.0258	HOMO → LUMO+3 (0.59)	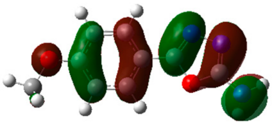 HOMO	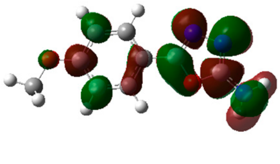 LUMO+3
S3	233.13	0.0046	HOMO → LUMO+1 (0.61)	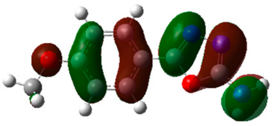 HOMO	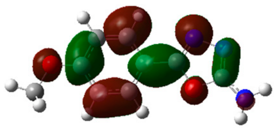 LUMO+1
S4	217.43	0.0024	HOMO → LUMO+2 (0.60)	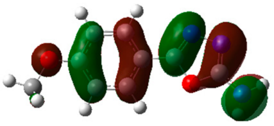 HOMO	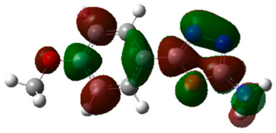 LUMO+2
S5	207.69	0.0041	HOMO-3 → LUMO (0.63)	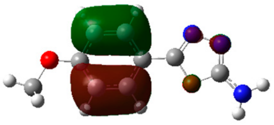 HOMO-3	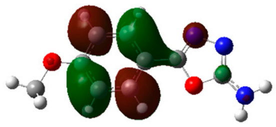 LUMO
S6	202.12	0.1328	HOMO-1 → LUMO (0.58)	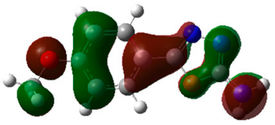 HOMO-1	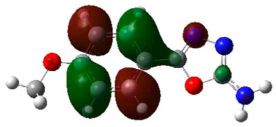 LUMO

Table S4. Excitation energies (λ, in nm), oscillator strengths (f), main orbital transitions (with corresponding amplitudes in parentheses) and isosurface representations of the orbitals involved for the six longest singlet states for the imino tautomer of 2-Amino-5-(4-methoxyphenyl)-1,3,4-oxadiazole **1′-(Z)s**, obtained from TD-DFT calculations at the CAM-B3LYP/6-311++G(d,p) level of theory.

State	*λ*	*f*	Major contribution	Donor orbital	Acceptor orbital
S1	266.64	0.5499	HOMO→LUMO (0.64)	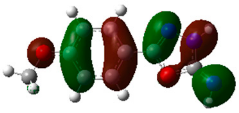<br>HOMO	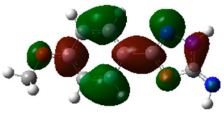<br>LUMO
S2	251.75	0.0127	HOMO→LUMO+2 (0.56)	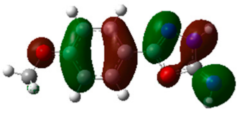<br>HOMO	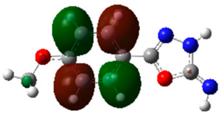<br>LUMO+2
S3	233.38	0.0017	HOMO→LUMO+3 (0.5)	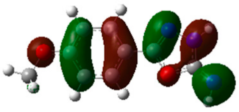<br>HOMO	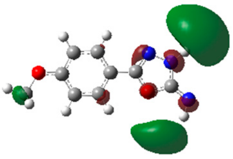<br>LUMO+3
S4	215.84	0.0051	HOMO→LUMO+1 (0.52)	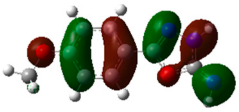<br>HOMO	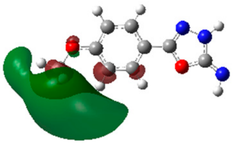<br>LUMO+1
S5	215.53	0.0422	HOMO-1→LUMO (0.66)	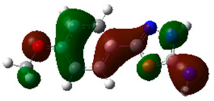<br>HOMO-1	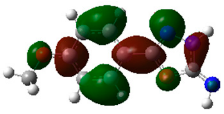<br>LUMO
S6	205.87	0.0009	HOMO→LUMO+4 (0.51)<br>HOMO→LUMO+6 (0.37)	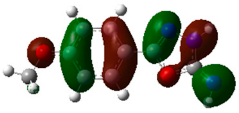<br>HOMO	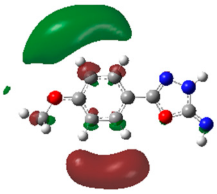<br>LUMO+4

				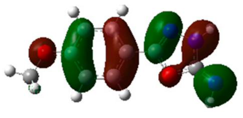<br>HOMO	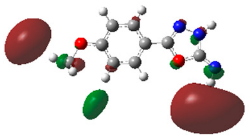<br>LUMO+6

Table S5. Excitation energies (λ, in nm), oscillator strengths (f), main orbital transitions (with corresponding amplitudes in parentheses) and isosurface representations of the orbitals involved for the six longest singlet states for the imino tautomer of 2-Amino-5-(4-methoxyphenyl)-1,3,4-oxadiazole **1′-(E)s**, obtained from TD-DFT calculations at the CAM-B3LYP/6-311++G(d,p) level of theory.

State	*λ*	*f*	Major contribution	Donor orbital	Acceptor orbital
S1	262.70	0.5451	HOMO→LUMO (0.69)	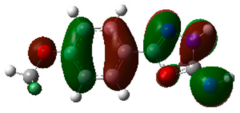<br>HOMO	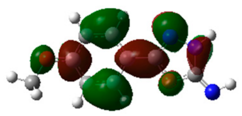<br>LUMO
S2	250.06	0.0128	HOMO→LUMO+3 (0.55)<br>HOMO-2→LUMO (0.33)	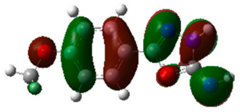<br>HOMO	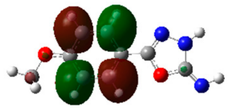<br>LUMO+3
				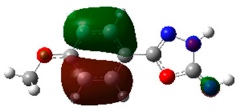<br>HOMO-2	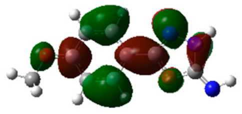<br>LUMO
S3	242.81	0.0012	HOMO→LUMO+1 (0.50)<br>HOMO→LUMO+2 (-0.39)	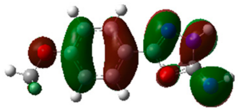<br>HOMO	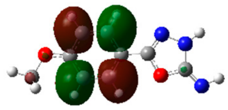<br>LUMO+3
				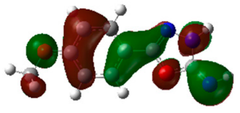<br>HOMO-1	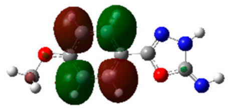<br>LUMO+3
S4	215.24	0.0372	HOMO-1→LUMO (0.65)	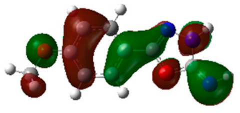<br>HOMO-1	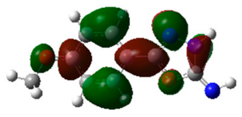<br>LUMO
S5	215.15	0.0067	HOMO→LUMO+2 (0.42)<br>HOMO→LUMO+1 (0.36)	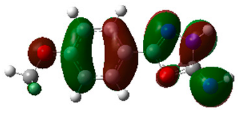<br>HOMO	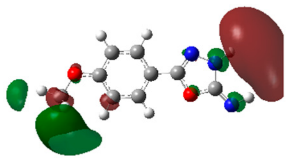

<table>
<tr><td rowspan="2"></td><td rowspan="2"></td><td rowspan="2"></td><td rowspan="2"></td><td></td><td>LUMO+2</td></tr>
<tr><td>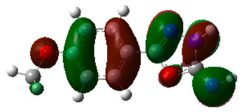
HOMO</td><td>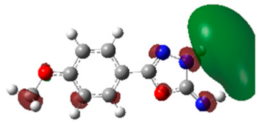
LUMO+1</td></tr>
<tr><td rowspan="2">S6</td><td rowspan="2">201.41</td><td rowspan="2">0.1042</td><td rowspan="2">HOMO-2→LUMO (0.53)
HOMO→LUMO+3 (-0.40)</td><td>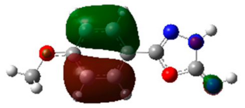
HOMO-2</td><td>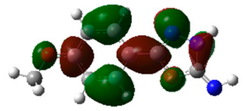
LUMO</td></tr>
<tr><td>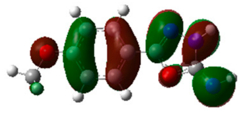
HOMO</td><td>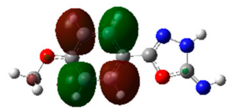
LUMO+3</td></tr>
</table>

3. Computational data

3.1. Cartesian coordinates of the optimized structures of the species investigated in this work. In cases where multiple conformers exist, only the lowest-energy conformer is reported.

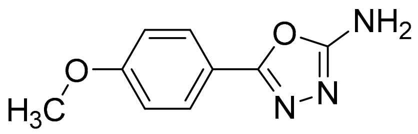

1′-a

	B3LYP/6-311++G(d,p)				B3LYP/aug-cc-pVTZ		
8	2.357304	0.730425	-0.003232	8	2.354044	-0.729197	0.001432
6	3.568070	0.119246	-0.006632	6	3.562354	-0.118234	0.004344
7	3.493875	-1.172896	0.000516	7	3.488254	1.171370	0.001013
7	2.126261	-1.469594	0.021084	7	2.120938	1.466977	-0.015535
6	1.487783	-0.347306	0.012083	6	1.484808	0.346518	-0.008921
6	0.055384	-0.098142	0.007209	6	0.054949	0.097362	-0.005159
6	-0.465389	1.206062	0.020933	6	-0.465426	-1.203220	-0.016398
6	-1.833483	1.419164	0.017289	6	-1.830113	-1.416453	-0.013733
6	-2.717150	0.332500	0.000712	6	-2.712510	-0.333140	-0.000742
6	-2.209071	-0.972063	-0.012296	6	-2.205095	0.968126	0.009711
6	-0.833903	-1.178326	-0.009344	6	-0.833678	1.173921	0.007806
8	-4.041720	0.644729	-0.001192	8	-4.035448	-0.643464	0.000518
6	-4.994872	-0.411838	-0.014103	6	-4.985508	0.412826	0.011241
7	4.679690	0.912222	0.051521	7	4.673168	-0.908832	-0.056222
1	0.207435	2.054410	0.036455	1	0.205175	-2.050224	-0.028905
1	-2.243733	2.421494	0.028447	1	-2.237067	-2.417633	-0.022803
1	-2.869713	-1.828176	-0.025077	1	-2.864689	1.821886	0.019644
1	-0.438057	-2.186358	-0.019729	1	-0.439911	2.180139	0.016137
1	-4.896070	-1.026801	-0.914722	1	-4.885468	1.028583	0.908155

1	-5.970808	0.070235	-0.011661	1	-5.961220	-0.064604	0.009222
1	-4.900345	-1.044690	0.874482	1	-4.889010	1.043436	-0.875668
1	5.539866	0.420879	-0.143617	1	5.531156	-0.420637	0.143445
1	4.603754	1.813868	-0.396151	1	4.597057	-1.812499	0.381488

MP2/6-311++G(d,p)

8	2.351083	-0.729406	0.104801
6	3.557501	-0.116483	0.055656
7	3.485513	1.175463	-0.118083
7	2.122117	1.461978	-0.206954
6	1.488273	0.328001	-0.063269
6	0.054532	0.083556	-0.020185
6	-0.467088	-1.217372	-0.149499
6	-1.843057	-1.423136	-0.124945
6	-2.723744	-0.333437	-0.022237
6	-2.208141	0.967276	0.096520
6	-0.824605	1.170069	0.087815
8	-4.050015	-0.645022	-0.027072
6	-4.961019	0.438123	0.104977
7	4.676910	-0.914814	0.085284
1	0.205044	-2.065320	-0.244295
1	-2.263276	-2.420399	-0.217547
1	-2.862922	1.826734	0.183433
1	-0.418242	2.173146	0.181931
1	-4.815149	0.963095	1.055465
1	-5.954260	-0.008576	0.083610
1	-4.859626	1.144524	-0.726236
1	5.519059	-0.368688	0.215010
1	4.613145	-1.687060	0.736073

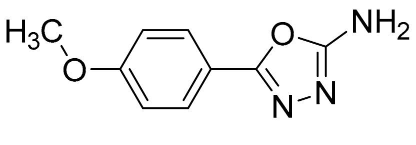

1′-s

B3LYP/6-311++G(d,p)				B3LYP/aug-cc-pVTZ			
8	-2.250005	-0.807533	0.003593	8	2.246454	-0.806372	-0.001642
6	-3.523196	-0.339816	0.004512	6	3.517420	-0.339273	-0.002628
7	-3.598543	0.952151	-0.003983	7	3.593240	0.950145	0.001740
7	-2.274267	1.404625	-0.022949	7	2.269362	1.402168	0.016992
6	-1.510597	0.363560	-0.011681	6	1.507702	0.362992	0.008666

6	-0.059239	0.283735	-0.004775	6	0.058824	0.283552	0.003228
6	0.608541	-0.942931	-0.019192	6	-0.609003	-0.939327	0.014875
6	2.001496	-1.001760	-0.013852	6	-1.998345	-0.997946	0.010832
6	2.744246	0.181601	0.005251	6	-2.739667	0.182234	-0.004165
6	2.079919	1.417911	0.019153	6	-2.075810	1.415098	-0.015233
6	0.699656	1.468483	0.014488	6	-0.699016	1.464938	-0.011844
8	4.103650	0.241104	0.011602	8	-4.097133	0.240433	-0.008944
6	4.842274	-0.974503	-0.003937	6	-4.832920	-0.974249	0.003407
7	-4.536146	-1.255560	-0.054401	7	4.529321	-1.253112	0.058551
1	0.040792	-1.864985	-0.036741	1	-0.043744	-1.860076	0.028969
1	2.488211	-1.967352	-0.026001	1	-2.484401	-1.961065	0.020583
1	2.672961	2.324163	0.033869	1	-2.665497	2.320807	-0.026684
1	0.186950	2.422250	0.025400	1	-0.188074	2.416874	-0.020500
1	5.891243	-0.683901	0.003964	1	-5.880773	-0.688031	-0.002624
1	4.633556	-1.556081	-0.908099	1	-4.622010	-1.557686	0.902847
1	4.627471	-1.582707	0.881108	1	-4.617482	-1.578615	-0.881041
1	-5.447460	-0.865335	0.137199	1	5.438252	-0.866528	-0.138459
1	-4.358774	-2.140818	0.397278	1	4.350591	-2.140684	-0.382217

MP2/6-311++G(d,p)

8	2.243272	-0.805356	-0.092165
6	3.513153	-0.338478	-0.035760
7	3.592619	0.953988	0.129664
7	2.271839	1.400142	0.202881
6	1.509755	0.347700	0.061451
6	0.057781	0.275775	0.006560
6	-0.612754	-0.949558	0.114197
6	-2.011968	-1.003211	0.072300
6	-2.749559	0.184584	-0.036694
6	-2.076163	1.415730	-0.135285
6	-0.687938	1.465419	-0.110404
8	-4.109540	0.253737	-0.072155
6	-4.814342	-0.973058	0.060935
7	4.530885	-1.263464	-0.050614
1	-0.045713	-1.870788	0.213483
1	-2.502855	-1.966288	0.153067
1	-2.668227	2.321810	-0.225945
1	-0.165721	2.414460	-0.189478
1	-5.871131	-0.711301	0.026126
1	-4.587164	-1.456910	1.017347
1	-4.579728	-1.656032	-0.763113
1	5.432523	-0.820605	-0.174443
1	4.382971	-2.025485	-0.699809

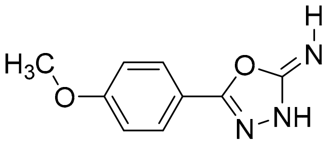

1″-(Z)s

B3LYP/6-311++G(d,p)

8	-2.235850	-0.857224	0.017669
6	-3.586268	-0.508024	0.001158
7	-3.557876	0.863004	0.017718
7	-2.278362	1.368179	-0.007620
6	-1.523492	0.324391	0.003163
6	-0.071244	0.272353	0.002863
6	0.604923	-0.949779	0.003147
6	1.998035	-0.997375	0.000930
6	2.731523	0.191889	-0.001554
6	2.057636	1.424056	-0.001558
6	0.677644	1.463690	0.000938
8	4.088971	0.262748	-0.003641
6	4.839015	-0.946975	-0.003088
7	-4.592116	-1.272620	-0.017825
1	-4.354924	1.468955	-0.069762
1	0.041679	-1.874410	0.005152
1	2.492231	-1.959146	0.001347
1	2.644211	2.334564	-0.002859
1	0.161048	2.415557	0.001828
1	5.885064	-0.646325	-0.004858
1	4.631083	-1.544337	-0.896866
1	4.633448	-1.542043	0.892762
1	-4.326962	-2.252302	-0.020869

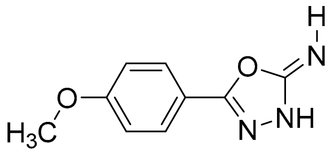

1″-(Z)a

B3LYP/6-311++G(d,p)

8	2.348796	0.777827	0.022482
6	3.649138	0.273355	0.001932
7	3.460980	-1.085259	0.020023
7	2.130733	-1.437056	-0.011384
6	1.503298	-0.312128	0.003355
6	0.066911	-0.087968	0.003419
6	-0.460775	1.213497	0.000844
6	-1.830036	1.417552	-0.001767
6	-2.706844	0.325227	-0.001683
6	-2.190763	-0.977145	0.001365
6	-0.814945	-1.174463	0.004314
8	-4.032152	0.628303	-0.004080
6	-4.980260	-0.433464	-0.004021
7	4.737267	0.915161	-0.020042
1	4.180785	-1.778546	-0.087022
1	0.209076	2.063953	0.000896
1	-2.246578	2.417253	-0.003672
1	-2.846555	-1.837001	0.002425
1	-0.416456	-2.181639	0.007886
1	-5.958328	0.044043	-0.006519
1	-4.881967	-1.055005	0.892018
1	-4.878811	-1.057935	-0.897670
1	4.588476	1.919144	-0.022284

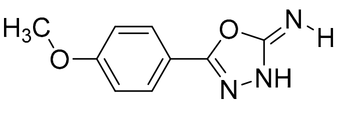

1″-(E)s

B3LYP/6-311++G(d,p)

8	-2.223265	-0.856226	0.045111
6	-3.568911	-0.529183	0.001332
7	-3.562444	0.852578	0.041796
7	-2.277893	1.368191	-0.023772
6	-1.523172	0.324685	0.007832

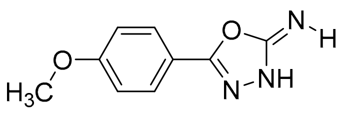

1″-(E)a

B3LYP/6-311++G(d,p)

8	2.336663	0.779200	0.049576
6	3.634793	0.297000	0.003568
7	3.466751	-1.074968	0.040265
7	2.130204	-1.435873	-0.029617
6	1.503180	-0.311166	0.006918

6	-0.070339	0.275045	0.008269
6	0.603669	-0.948360	0.004807
6	1.996427	-0.996623	-0.000963
6	2.730682	0.192435	-0.003032
6	2.058360	1.425481	0.001624
6	0.678295	1.465998	0.008153
8	4.088014	0.261904	-0.008070
6	4.836977	-0.948739	-0.010606
7	-4.462218	-1.422751	-0.035160
1	-4.333204	1.444753	-0.217699
1	0.038037	-1.871547	0.006267
1	2.489916	-1.958720	-0.003412
1	2.646003	2.335284	0.001928
1	0.162098	2.418104	0.014172
1	5.883274	-0.648960	-0.013814
1	4.626763	-1.544544	-0.904817
1	4.632314	-1.544812	0.884708

6	0.065900	-0.089397	0.007501
6	-0.460264	1.212724	-0.003389
6	-1.829351	1.416414	-0.009613
6	-2.706069	0.323779	-0.004663
6	-2.190818	-0.978927	0.007663
6	-0.814944	-1.176147	0.014802
8	-4.031190	0.626897	-0.010957
6	-4.979669	-0.434426	-0.005540
7	4.626595	1.079705	-0.030743
1	4.161722	-1.750567	-0.229550
1	0.211234	2.061898	-0.007258
1	-2.245961	2.416028	-0.018008
1	-2.847026	-1.838421	0.014112
1	-0.416132	-2.183162	0.027254
1	-5.957539	0.043452	-0.012739
1	-4.883098	-1.050153	0.894700
1	5.508640	0.575737	-0.894937

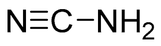

2

B3LYP/6-311++G(d,p)

7	1.378598	0.000001	0.012281
6	0.220906	-0.000003	-0.000774
7	-1.117297	0.000000	-0.076325
1	-1.577266	0.847654	0.226476
1	-1.577277	-0.847645	0.226478
7	1.378598	0.000001	0.012281
6	0.220906	-0.000003	-0.000774

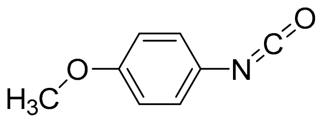

3

B3LYP/6-311++G(d,p)

7	2.420820	0.584738	0.000000
6	1.056402	0.270189	0.000000
6	0.128965	1.308713	0.000000
6	-1.239899	1.040802	0.000000
6	-1.690453	-0.281343	0.000000
6	-0.757655	-1.327016	0.000000
6	0.600254	-1.056523	0.000000
8	-3.001641	-0.655577	0.000000
6	-3.998486	0.358127	0.000000
6	3.501068	0.058377	0.000000
8	4.603374	-0.340233	0.000000
1	0.482960	2.332200	0.000000
1	-1.935631	1.868677	0.000000
1	-1.120892	-2.347458	0.000000
1	1.313884	-1.872637	0.000000
1	-3.929149	0.986352	-0.894641
1	-3.929149	0.986352	0.894641
1	-4.953397	-0.164495	0.000000

$O=C=N-NH_2$

4

B3LYP/6-311++G(d,p)

7	0.443215	-0.444430	-0.000012
6	-0.701877	-0.042301	0.000000
8	-1.853851	0.155000	0.000004
7	1.654883	0.308145	0.000012
1	2.177682	-0.016057	-0.811155
1	2.177701	-0.016143	0.811131

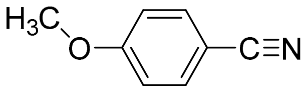

5

B3LYP/6-311++G(d,p)

6	2.965658	-0.179731	-0.000006
6	1.545432	-0.026363	0.000000
6	0.711584	-1.149771	0.000010
6	-0.672387	-1.007779	0.000015
6	-1.238358	0.272476	0.000010
6	-0.406686	1.402714	0.000000
6	0.967468	1.256320	-0.000002
8	-2.571103	0.523263	0.000020
6	-3.480668	-0.573741	-0.000026
7	4.115485	-0.302499	-0.000010
1	1.149227	-2.140544	0.000014
1	-1.292965	-1.893100	0.000024
1	-0.865232	2.383711	-0.000003
1	1.606399	2.130797	-0.000009
1	-3.357185	-1.191388	0.895206
1	-3.357158	-1.191328	-0.895297
1	-4.474910	-0.131507	-0.000025

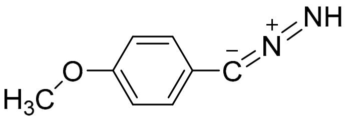

6A

B3LYP/6-311++G(d,p)

6	-2.408751	-0.591787	-0.032478
6	-1.005248	-0.283429	-0.016394
6	-0.547283	1.051891	-0.017077
6	0.804682	1.334911	-0.010158
6	1.744990	0.292170	0.000898
6	1.308458	-1.036790	0.001499
6	-0.056127	-1.312177	-0.010556
8	3.047637	0.675345	0.009333
6	4.059936	-0.326245	0.017289
7	-3.494952	-0.117991	0.036719
7	-4.673384	0.284527	-0.107443
1	-1.267178	1.861898	-0.026147
1	1.164681	2.356587	-0.012419
1	2.013748	-1.856407	0.007727
1	-0.396749	-2.340494	-0.015908
1	4.003619	-0.955070	-0.877090
1	3.992298	-0.952458	0.912757
1	5.006396	0.210987	0.022488
1	-5.123502	0.335187	0.810860

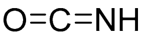

7

B3LYP/6-311++G(d,p)

8	-1.210331	0.013970	-0.000005
6	-0.044846	0.021688	0.000014
7	1.158142	-0.122204	-0.000004
1	1.844734	0.613537	-0.000008
8	-1.210331	0.013970	-0.000005
6	-0.044846	0.021688	0.000014
7	1.158142	-0.122204	-0.000004
1	1.844734	0.613537	-0.000008

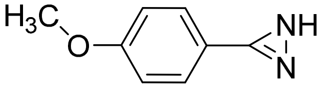

8

B3LYP/6-311++G(d,p)

6	0.871079	1.437251	0.020043
6	-0.505955	1.352508	0.011938
6	-1.138962	0.095493	-0.007643
6	-0.359307	-1.063519	-0.022255
6	1.030425	-0.987651	-0.016091
6	1.650802	0.266883	0.005998
1	1.377360	2.394452	0.035256
1	-1.107539	2.254046	0.020734

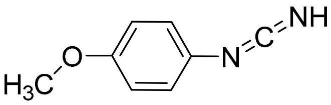

9

B3LYP/6-311++G(d,p)

6	-0.709990	1.310958	-0.017421
6	0.635122	0.983404	-0.026802
6	1.039417	-0.360936	-0.018847
6	0.067013	-1.358112	-0.003968
6	-1.290020	-1.035459	0.006869
6	-1.685057	0.304256	0.000567
1	-1.031995	2.345268	-0.025241
1	1.380980	1.770520	-0.045548

1	-0.847601	-2.030980	-0.049074	1	0.380772	-2.394857	-0.001714
1	1.612682	-1.898476	-0.031310	1	-2.019645	-1.833649	0.018636
8	2.992154	0.459453	0.014427	8	-2.980382	0.732709	0.009469
6	3.854589	-0.675242	-0.004367	6	-4.017497	-0.239017	0.021868
1	4.866597	-0.275512	0.004944	1	-4.950465	0.322024	0.025388
1	3.702759	-1.302985	0.879313	1	-3.980227	-0.874641	-0.869517
1	3.706295	-1.270410	-0.910835	1	-3.967887	-0.865179	0.919370
6	-2.574658	-0.010036	-0.015382	7	2.391506	-0.750245	-0.035120
7	-3.644938	0.632194	-0.045916	6	3.417455	-0.100779	0.024447
7	-3.560458	-1.023626	-0.067517	7	4.502398	0.460279	-0.075874
1	-3.778088	-1.299854	0.896143	1	5.035536	0.672711	0.759544
